# *Giardia duodenalis* MIF induces host intestinal damage via CD74 receptor mediated NLRP3 inflammasome activation

**DOI:** 10.1371/journal.pntd.0013968

**Published:** 2026-02-02

**Authors:** Mengge Chen, Jianhua Li, Xiaocen Wang, Zhenzhen Liu, Xu Zhang, Heng Yang, Xuancheng Zhang, Hongyu Wang, Hongyan Kang, Yanhui Yu, Pengtao Gong, Nan Zhang, Xin Li

**Affiliations:** 1 State Key Laboratory for Diagnosis and Treatment of Severe Zoonotic Infectious Diseases, Key Laboratory for Zoonosis Research of the Ministry of Education, Institute of Zoonosis, and College of Veterinary Medicine, Jilin University, Changchun, China; 2 Second Affiliated Hospital, Jilin University, Changchun, China; Georgetown University, UNITED STATES OF AMERICA

## Abstract

*Giardia duodenalis* is an important zoonotic protozoan that mainly causes diarrhea, has a significant negative impact on public health worldwide. Macrophage migration inhibitory factor (MIF) as an inflammatory mediator in both innate and adaptive immune responses, and parasite-derived MIF is involved in inducing the host’s immune response or causing disease. However, the role of *G. duodenalis* MIF (GdMIF) in giardiasis remains to be elucidated. In the present study, CD74-NF-κB-NLRP3 inflammasome activation induced by rGdMIF was systematically investigated *in vitro* and *in vivo*, and its effect on intestinal damage was examined in *G. duodenalis-*infected gerbils. We found that GdMIF was an exocrine protein with dopamine tautomerase activity. GdMIF could activate NF-κB and the NLRP3 inflammasome, increase GSDMD-processing and promote Lactate Dehydrogenase (LDH) and pro-inflammatory cytokine release. The interaction of CD74 molecule with rGdMIF was validated by Co-IP and BiFC. Furthermore, knockdown of CD74 and NF-κB significantly inhibited NLRP3 inflammasome activation and pro-inflammatory cytokine production in macrophages stimulated by rGdMIF. Gerbils were infected with *G. duodenalis* in the presence of a GdMIF blocking antibody showed lower NLRP3 expression, and milder intestinal damage compared with that of the normal *G. duodenalis* infection group. Inhibition of NLRP3 alleviated intestinal damage caused by *G. duodenalis* infection. In summary, these findings suggest that GdMIF induces NLRP3 inflammasome activation and pyroptosis by interacting with CD74 receptor, subsequently eliciting a pro-inflammatory response which lead to intestinal damage.

## Author summary

*Giardia duodenalis* is a globally important protozoan parasite that infects the small intestine and causes diarrheal disease. The mammalian macrophage migration inhibitory factor (MIF) is a critical host cytokine governing immune regulation and inflammatory responses. GdMIF is a homolog of mammalian MIF, however, its roles in *G. duodenalis* infection remain unknown. In this study, we demonstrated that GdMIF is secreted by *G. duodenalis* and possesses dopachrome tautomerase activity. GdMIF specifically binds to the host receptor CD74 on macrophages, triggering the NF-κB signaling cascade and subsequent activation of the NLRP3 inflammasome. This cascade caused pyroptosis and robust secretion of pro-inflammatory cytokines (IL-1β, IL-6, IL-12, IL-18, and TNF-α). In a gerbil model of giardiasis, blocking GdMIF with specific antibodies or pharmacologically inhibiting NLRP3 significantly reduced intestinal inflammation, preserved goblet cells and tight-junction integrity, and alleviated villous damage. These findings reveal a novel mechanism by which *G. duodenalis* utilizes its own MIF homologue to exacerbate host intestinal injury via the CD74-NF-κB-NLRP3 axis. Targeting GdMIF or NLRP3 may offer new therapeutic avenues for preventing or treating giardiasis and its associated morbidities.

## 1. Introduction

*Giardia duodenalis* (*G. duodenalis*) is a globally distributed zoonotic protozoan that infects the upper small intestine, leading to acute watery diarrhea in approximately 280 million individuals annually [1]. *G. duodenalis* infection causes malnutrition and stunted growth in children [2]. *G. duodenalis* attaches to the duodenal epithelial cells and causes intestinal mucosal damage, absorption dysfunction, and intestinal flora imbalance through ventral disc adsorption, nutritional competition, leading to diarrhea, abdominal pain, and weight loss. Metronidazole and tinidazole are commonly used in giardiasis treatment, however, drug resistance and drug-induced teratogenicity and mutation pose serious concerns. In addition, there is no effective prevention vaccines for giardiasis [3].

Macrophage migration inhibitory factor (MIF) is a pleiotropic cytokine initially discovered in T cells, involved in regulating their activation and proliferation [4]. Later research has demonstrated that human MIF (HuMIF) inhibits macrophage random migration, enhancing their retention and function at inflammatory sites. Moreover, HuMIF plays important roles in immune regulation, inflammatory response and cell proliferation [5–7]. In addition, many studies have found that HuMIF is highly expressed in tumors. HuMIF is believed to promote tumor growth by influencing cell proliferation and invasiveness [8–10].

The pyrin domain containing 3 (NLRP3) inflammasome, a multi-protein complex comprising the NOD-like receptor family member NLRP3, ASC and pro‐caspase-1, is crucial for innate immunity, regulating inflammation and pyroptosis [11]. The NLRP3 inflammasome plays important roles in multiple diseases, including infectious diseases, autoimmune and metabolic diseases [12]. There is a close functional link between MIF and NLRP3 inflammasome. It has been found that MIF can induce the activation of NLRP3 inflammasome [7]. The mouse MIF inhibitor ISO-1 mitigated NF-κB and NLRP3 pathway activation in the kidney and liver, reducing organ damage and dysfunction in an acute hemorrhagic shock model [13].

CD74 is a type II transmembrane glycoprotein found on various cell types such as B cells, macrophages, endothelial cells, and cancer cells [5,14–17]. CD74 is not only an accessory molecule of MHC-II, but also a high-affinity receptor for MIF [18,19]. The interaction between mammalian MIF and CD74 is crucial for immune regulation, cell signaling, and inflammatory responses, contributing to the pathogenesis of infectious diseases, autoimmune and cancer diseases [20–22].

Notably, MIF is not only present in mammalian cells, but homologues of MIF have also been found in parasites and plants [23–26]. Recent studies have found that a variety of parasites, such as *Toxoplasma gondii*, *Trichomonas vaginalis*, *Plasmodium*, and *Leishmania*, can secrete MIF homologues, which are structurally similar to HuMIF protein [27–30]. *Leishmania* MIF promotes the random migration of host monocytes, thereby attenuating their recruitment at sites of inflammation and providing a favorable environment for the replication and development of the intracellular protozoa themselves. *P. yoelii* MIF enhances IFN-γ and IL-6 secretion in mice, while also recruits and activates monocytes [29]. *T. vaginalis* MIF and *T. gondii* MIF can activate ERK and Akt related inflammatory pathways in macrophages [31,32]. *T. gondii* MIF triggers the NLRP3 inflammasome, leading to IL-1β and IL-18 release via hepatocyte pyroptosis, inhibiting parasite reproduction, and causing severe liver injury during *T. gondii* infection [32]. In addition, it has been found that *Plasmodium* MIF and *E. histolytica* MIF can also bind to CD74 in host cells [33,34]. *G. duodenalis* MIF (GdMIF) has been found to share a high structural similarity with HuMIF protein [35]. However, the interaction of GdMIF with host cells and the potential role of GdMIF in giardiasis need to be explored.

In this study, the rGdMIF protein was expressed and its distribution in *G. duodenalis* and dopachrome tautomerase activity were analyzed. Furthermore, the expression changes of NOD-like genes were examined in mouse macrophages stimulated by rGdMIF. NLRP3 inflammasome activation and pyroptosis, NF-κB pathway induced by rGdMIF were further investigated. In addition, the interaction of CD74 with rGdMIF protein was validated. The effects of GdMIF and NLRP3 on intestinal damage were evaluated in *G. duodenalis-*infected gerbils.

## 2. Results

### 2.1. *G. duodenalis* secretes GdMIF protein and rGdMIF has dopamine tautomerase activity

**Fig 1:**
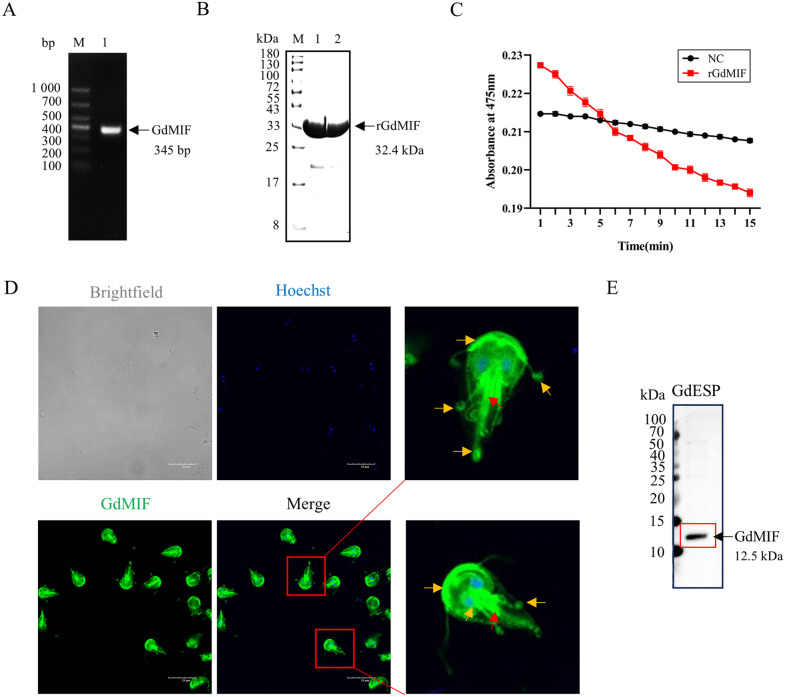
Preparation of rGdMIF and localization of GdMIF. (A) *GdMIF* gene was amplified by PCR, and the expected size for the PCR product was 345 bp. (B) Purification of rGdMIF protein. Lanes 1 and 2 represented different batches of rGdMIF, the expected molecular weight for the rGdMIF was 32.4 kDa. (C) Testing for the D-dopachrome tautomerase activity by rGdMIF. (D) The localization of GdMIF in *G. duodenalis* was observed by performing an IFA and imaged with confocal microscopy. Hoescht staining was utilized to stain the nuclear DNA (blue). GdMIF was detected using the polyclonal antibodies against rGdMIF (green). Scale bar = 20 um. *G. duodenalis* was zoomed in image. (E) GdMIF protein in GdESP was detected by western blot, the polyclonal antibodies against rGdMIF was utilized to detect GdMIF. All results were representative of three number of biologically independent experiments (n = 3).

To explore the function and subcellular localization of GdMIF, the GdMIF gene was successfully amplified, yielding a product of 345 bp (Fig 1A). A recombinant pET32a-GdMIF plasmid was constructed for protein expression. SDS-PAGE analysis confirmed the expression and purification of soluble rGdMIF with a molecular weight of 32.4 kDa (Fig 1B). To verify whether the purified rGdMIF retained enzymatic activity analogous to the native GdMIF protein, we assessed its dopamine tautomerase activity. The results demonstrated that rGdMIF possessed significant dopamine tautomerase activity (Fig 1C), indicating its functional competence for subsequent studies. Polyclonal antibody against rGdMIF were then generated in mice, and the antibody is played the specificity of GdMIF (S1 Fig). IFA using these antibodies localized GdMIF to the axial columns and flagella of *G. duodenalis* trophozoites (Fig 1D). Furthermore, western blot analysis revealed that GdMIF protein also existed in GdESP (Fig 1E).

### 2.2. GdMIF regulates NLRP3 inflammasome activation and pyroptosis in mouse PMϕs

**Fig 2:**
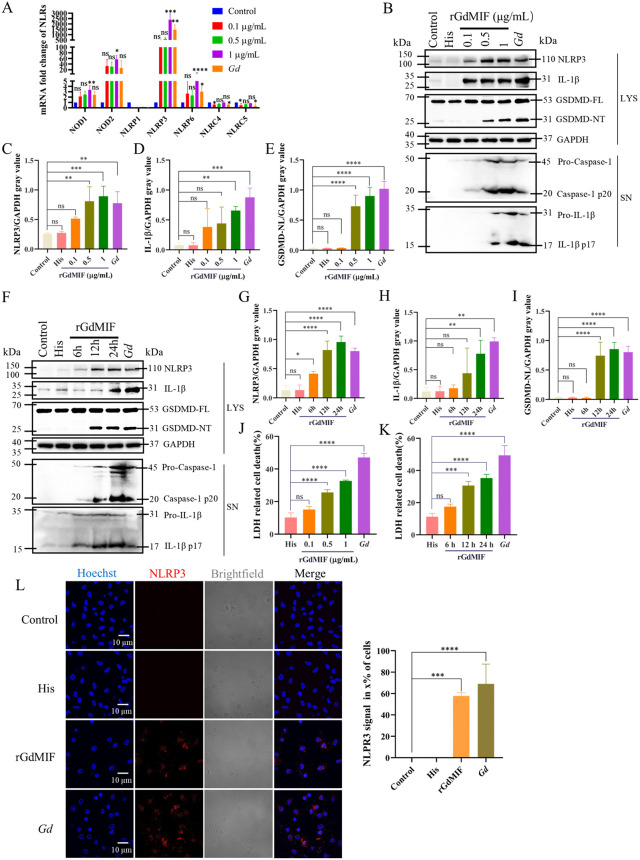
rGdMIF regulates NLRP3 inflammasome activation and pyroptosis in PMϕs. (A) 0.1 μg/mL, 0.5 μg/mL or 1 μg/mL rGdMIF and *G. duodenalis* (MOI = 3) were individually added into PMϕs for 24 h, and the mRNA expression levels of NODs in PMϕs were measured by qPCR assays. (B-I) PMϕs were incubated with 0.1 μg/mL, 0.5 μg/mL or 1 μg/mL rGdMIF for 24 h, or were incubated with 1 μg/mL rGdMIF or His for 6, 12, 24 h, respectively. The protein expression of NLRP3, IL-1β, GSDMD-FL and GSDMD-NT, Caspase-1 p20 and IL-1β p17 were detected using western blot and densitometric analysis. (J. K) Cell death was detected by LDH assay. (L) PMϕs were incubated with 1 μg/mL His, 1 μg/mL rGdMIF, or *G. duodenalis* (MOI = 3) for 24 h, NLPR3 (red) was detected by IFA, scale bar: 10 μm, and three images of view were captured in total per sample, quantitative analysis of the presence of NLPR3 signals in x% of the cells in each image was performed using Image J software. All results were representative of three number of biologically independent experiments (n = 3). Data was shown as the mean ± SD, statistical significance was assessed using one-way ANOVA and unpaired Student’s t-test. n.s. *p* > 0.05 indicates not significant, *p < 0.05, **p < 0.01, ***p < 0.001 and ****p < 0.0001.

To determine whether GdMIF participates in the inflammatory response, we first examined the expression of NODs pattern recognition receptor in mouse PMϕs treated with rGdMIF. The mRNA expression levels of NOD1 (*p* = 0.0041), NOD2 (*p* = 0.0133), NLRP3 (*p* < 0.001) and NLRP6 (*p* < 0.0001) were significantly increased, and NLRP3 expression was the highest up-regulated both in the rGdMIF-treated group and the *G*. *duodenalis*-treated group compared with the untreated mouse PMϕs (Control) group (Fig 2A). The rGdMIF treated group showed significant increase in NLRP3 inflammasome-related proteins (NLRP3, IL-1β, Caspase1 p20, and IL-1β p17) and the GSDMD-NT fragment compared to both the control and His-tag protein control groups (Fig 2B–I). Moreover, rGdMIF also promoted LDH release in concentration and time-dependent manner (Fig 2J and 2K) (*p* < 0.0001). Based on the dose-response and time-course experiments, which indicated that 1 μg/mL rGdMIF for 24 h induced the most significant NLRP3 inflammasome activation and pyroptosis (Fig 2B–K), this condition was selected for all subsequent mechanistic studies. The IFA results indicated that strong NLPR3 signal was observed in 57% of cells in the rGdMIF group, and in 69% of cells in the *G*. *duodenalis* group, while nonexistent in the control group and the His-tag protein control group (Fig 2L). These data suggest that rGdMIF activates the NLRP3 inflammasome and induces pyroptosis in mouse PMϕs.

### 2.3. GdMIF interacts with mouse CD74

**Fig 3:**
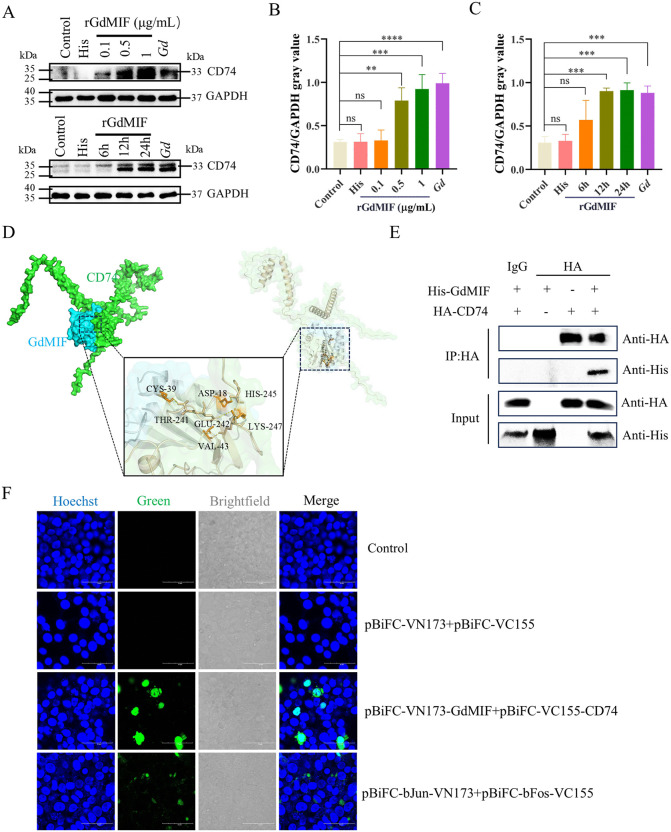
The analysis of GdMIF interactes with mouse CD74. (A) PMϕs were incubated with 0.1 μg/mL, 0.5 μg/mL, 1 μg/mL rGdMIF or *G. duodenalis* (MOI = 3) for 24 h. PMϕs were incubated with 1 μg/mL rGdMIF or His for 6, 12, 24 h, respectively. The protein expression of CD74 was detected using western blot. (B. C) The densitometric analysis of CD74 protein. The results were representative of three number of biologically independent experiments (n = 3), data was shown as the mean ± SD, statistical significance was assessed using one-way ANOVA. n.s. *p*> 0.05 indicates not significant, **p < 0.01, ***p < 0.001 and ****p < 0.0001. (D) The molecular docking model of GdMIF (blue) with CD74 (green) protein. (E) Co-IP assays of GdMIF and CD74. (F) BiFC experiments of GdMIF and CD74. (Blue: cell nuclei. Green: protein interactions. Scale bar: 50 μm). All results were representative of three number of biologically independent experiments (n = 3).

The interaction between mammalian MIF and CD74 is crucial for immune regulation, cell signaling, and inflammatory responses. To investigate whether GdMIF activated the NLRP3 inflammasome and induced pyroptosis through a related mechanism, we first examined CD74 expression in mouse PMϕs stimulated with rGdMIF. As shown in Fig 3A–C, rGdMIF treatment significantly up-regulated CD74 protein levels in a concentration- and time-dependent manner compared with the control group. To further explore the potential physical interaction between GdMIF and CD74, we performed molecular docking using AlphaFold and Pymol. The result showed that the computational model revealed a stable binding interface, and its binding energy was -18.2 kcal/mol. GdMIF docked well with mouse CD74 (Fig 3D), and GdMIF also docked well with human CD74, and its binding energy was -14.3 kcal/mol (S2 Fig), supporting the possibility of direct recognition between GdMIF and mouse/human CD74. Subsequently, we verified GdMIF protein binding to CD74 protein by Co-IP and BiFC assays. The Co-IP demonstrated successful expression of His-tagged protein in the pcDNA3.1-His-GdMIF group, HA-tagged protein in the pcDNA3.1-N-HA-CD74 group, and both tag proteins in the co-transfected group with the pcDNA3.1-His-GdMIF and pcDNA3.1-N-HA-CD74 plasmids, confirming the expression of GdMIF and CD74 proteins in HEK293T cells (Fig 3E). Following the collected protein samples, anti-HA antibody and protein A/G magnetic beads were co-incubated, and samples analyzed by western blot. The findings indicated an interaction between GdMIF and CD74 in eukaryotic cells (Fig 3E). In BiFC assay, green fluorescence was detected in the pBiFC-CD74-VC155 + pBiFC-GdMIF-VN173 group and the pBiFC-bFos-VC155 + pBiFC-bJun-VN173 group, but not in the negative control group and the pBiFC-VC155 + pBiFC-VN173 group (Fig 3F). These results prove the direct interaction between GdMIF and mouse CD74.

### 2.4. GdMIF activates NF-κB signal-mediated NLRP3 inflammasome and pyroptosis via CD74 receptor

**Fig 4:**
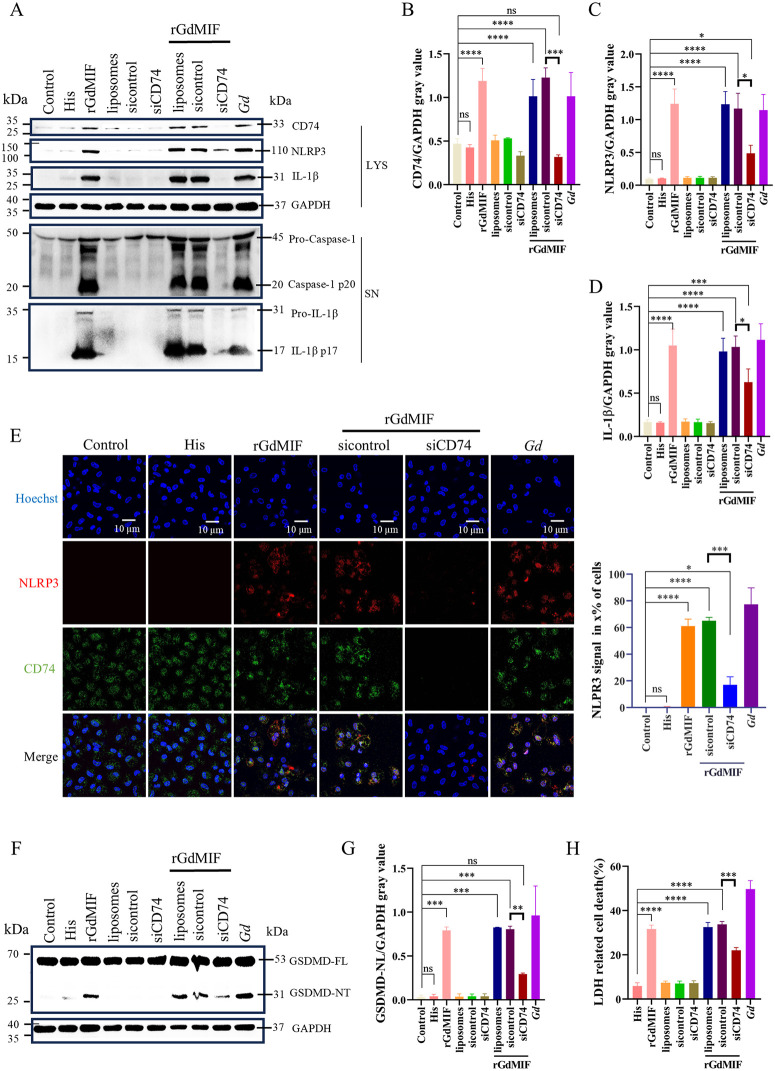
CD74 is involved in the NLRP3-GSDMD pathway activation induced by rGdMIF. PMϕs were transfected with control-siRNA (sicontrol) and CD74-siRNA (siCD74) for 24 h, and then stimulated with rGdMIF (1 μg/mL) for 24 h. (A-D) The protein expression of CD74, NLRP3, IL-1β, Caspase1 p20 and IL-1β p17 were detected by western blot and densitometric analysis. (E) NLRP3 (Red) and CD74 (green) were observed by IFA, scale bar: 10 μm. Three fields of view were analyzed in total per sample, and the quantitative analysis of NLPR3 signals in x% of the cells in each image was performed using Image J software. (F, G) The expression of GSDMD-FL and GSDMD-NT protein were detected by western blot and densitometric analysis. (H) Cell death was detected by LDH assay. All results were representative of three number of biologically independent experiments (n = 3). Data was shown as the mean ± SD, statistical significance was assessed using one-way ANOVA and unpaired Student’s t-test. n.s. *p* > 0.05 indicates not significant, *p < 0.05, **p < 0.01, ***p < 0.001 and ****p < 0.0001.

To clarify whether GdMIF regulated NLRP3 inflammasome activation via interaction with CD74, activation of the NLRP3 inflammasome and pyroptosis pathways were probed for after knocking down CD74 by siRNA. The results showed that liposomes only, control-siRNA (sicontrol), and CD74-siRNA (siCD74) did not significantly alter NLRP3, IL-1β, IL-1β p17, and caspase-1 p20 levels compared to the control group. The levels of CD74 (*p* = 0.0002), NLRP3 (*p* = 0.0108), IL-1β (*p* = 0.0236), IL-1β p17, and caspase-1 p20 proteins were significantly decreased (Fig 4A–D), and the NLRP3 signal in cells reduced 48% (*p* = 0.0002) (Fig 4E) in the siCD74 + rGdMIF group compared with the sicontrol + rGdMIF group. Meanwhile, adding the liposome, sicontrol, or siCD74 alone did not affect the protein level of GSDMD-NT, knocking down CD74 reduced GSDMD-NT fragment (*p* = 0.0024) (Fig 4F and 4G) and LDH release (*p* = 0.0003) (Fig 4H). These results indicate that rGdMIF activates NLRP3 inflammasome and induces pyroptosis by interacting with CD74.

**Fig 5:**
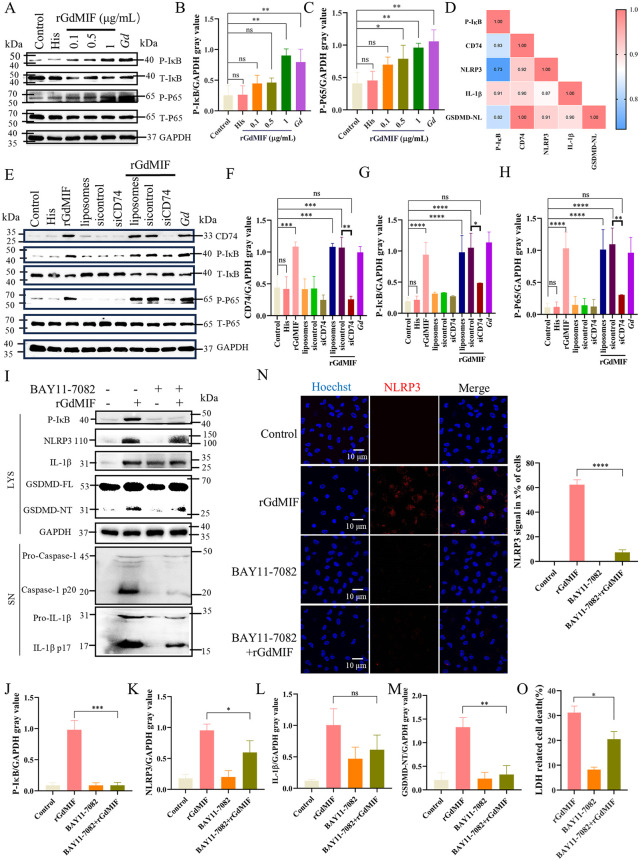
GdMIF regulates NLRP3-GSDMD activation through the CD74-NF-κB pathway. (A) PMϕs were incubated with 0.1 μg/mL, 0.5 μg/mL, 1 μg/mL rGdMIF or *G. duodenalis* (MOI = 3) for 24 h. The expression of total and phosphorylated proteins of IκB and P65 were detected by western blot. (B. C) The densitometric analysis of P-IκB and P-P65 protein. All results were representative of three number of biologically independent experiments (n = 3). Data was shown as the mean ± SD, statistical significance was assessed using one-way ANOVA. n.s. *p* > 0.05 indicates not significant, *p < 0.05, **p < 0.01. (D) Spearman correlation between P-IκB, CD74, NLRP3, IL-1β and GSDMD-NT. (weak correlation: 0.1 < r < 0.3, moderate correlation: 0.3 < r < 0.5, strong correlation: 0.5 < r < 1.0). (E-H) PMϕs were transfected with control-siRNA (sicontrol) and CD74-siRNA (siCD74) for 24 h, and then stimulated with rGdMIF (1 μg/mL) for 24 h. The expression of CD74, total and phosphorylated proteins of IκB and P65 were detected by western blot and densitometric analysis. All results were representative of three number of biologically independent experiments (n = 3). Data was shown as the mean ± SD, statistical significance was assessed using one-way ANOVA and unpaired Student’s t-test. n.s. *p* > 0.05 indicates not significant, *p < 0.05, ***p < 0.001 and ****p < 0.0001. (I-M) PMϕs were pretreated with BAY11-7082 (5 μM) for 2 h and then stimulated with rGdMIF (1 μg/mL) for 24 h. The expression levels of P-IκB, NLRP3-GSDMD pathway related proteins were detected by western blot and densitometric analysis. (N) NLRP3 (Red) was observed by IFA, scale bar: 10 μm. Three fields of view were analyzed in total per sample, and three images of view were captured in total per sample, quantitative analysis of the presence of NLPR3 signals in x% of the cells in each image was performed using Image J software. (O) Cell death was detected by LDH assay. All results were representative of three number of biologically independent experiments (n = 3). Data was shown as the mean ± SD, statistical significance was assessed using unpaired Student’s t-test. n.s. *p* > 0.05 indicates not significant, *p < 0.05, **p < 0.01, ***p < 0.001 and ****p < 0.0001.

To clarify whether CD74 regulated NF-κB pathway to further induce NLRP3 inflammasome activation, we tested for differential detection of NF-κB pathway proteins after knocking down CD74. The result found that rGdMIF stimulation significantly increased phosphorylated-IκBα (P-IκBα) and P-P65 levels in PMϕs (Fig 5A–C). We conducted a correlation analysis on the expression levels of related proteins in the CD74, NF-κB, NLRP3 and pyroptosis pathways induced by rGdMIF. The result showed that P-IκBα, CD74, NLRP3, IL-1β and GSDMD-NT were significantly positively correlated (Fig 5D). The levels of CD74, P-IκBα, and P-P65 proteins were unchanged in the liposome, sicontrol, or siCD74 groups compared with the control group, the levels of CD74 (*p* = 0.0012), P-IκBα (*p* = 0.0136), and P-P65 (*p* = 0.0056) proteins were significantly decreased in the siCD74 + rGdMIF group compared with the sicontrol + rGdMIF group (Fig 5E–H). Subsequently, NLRP3 inflammasome and pyroptosis pathway were detected in PMϕs after inhibiting IκB. The study found the expressions of P-IκBα (p = 0.0006), NLRP3 (*p* = 0.0449), IL-1β (*p* = 0.1241), and GSDMD-NT (*p* = 0.0033), cleaved IL-1β p17 and caspase-1 p20 fragment (Fig 5I–M) were significantly reduced, and NLRP3 signal in cells reduced 55% (*p* < 0.0001) (Fig 5N) in the BAY11–7082 + rGdMIF group compared to the rGdMIF group. The release of LDH was also reduced in the BAY11–7082 + rGdMIF group compared with the rGdMIF group (*p* = 0.0043) (Fig 5O). These data suggest that rGdMIF induces NLRP3 inflammasome and pyroptosis via NF-κB pathway.

**Fig 6:**
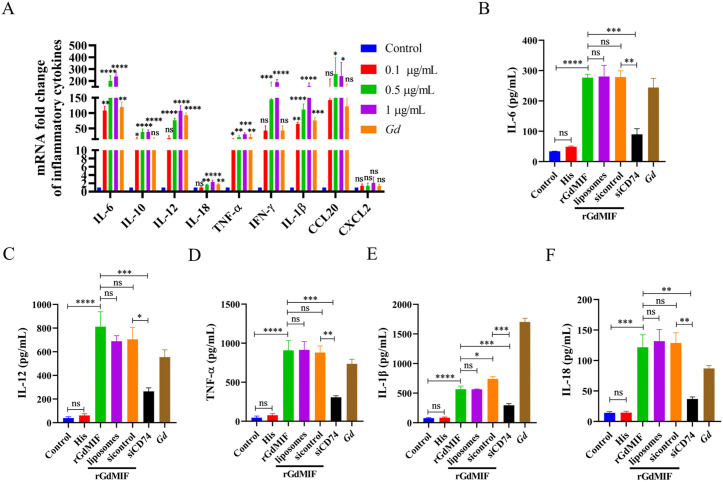
rGdMIF induces pro-inflammatory cytokines secretion by interacting with CD74. (A) PMϕs were incubated with 0.1 μg/mL, 0.5 μg/mL, 1 μg/mL rGdMIF for 24 h, and *G. duodenali* (MOI = 3) stimulated PMϕs as a positive control, and the mRNA expression levels of cytokines were detected by qPCR. Data are presented as mean ± SD from three independent experiments (n = 3), statistical significance was assessed using one-way ANOVA. n.s. *p* > 0.05 indicates not significant, *p < 0.05, **p < 0.01, ***p < 0.001 and ****p < 0.0001. (B-F) PMϕs were transfected with sicontrol and siCD74 for 24 h, then stimulated with rGdMIF (1 μg/mL) for 24 h. All results were representative of three number of biologically independent experiments (n = 3). Data was shown as the mean ± SD, statistical significance was assessed using one-way ANOVA and unpaired Student’s t-test. n.s. *p* > 0.05 indicates not significant, *p < 0.05, **p < 0.01, ***p < 0.001 and ****p < 0.0001.

The transcriptional levels of cytokines were detected by qPCR. The results showed that the transcriptional levels of IL-6, IL-10, IL-12, IL-18, TNF-α, IFN-γ and IL-1β were markedly elevated in the rGdMIF group compared with the control group (Fig 6A). Then ELISA results also indicated that rGdMIF enhanced the secretion of IL-6, IL-12, TNF-α, IL-1β, and IL-18 (Fig 6B–F), while the secretion of IL-6 (*p* = 0.0024), IL-12 (*p* = 0.0144), TNF-α (*p* = 0.0026), IL-1β (*p* = 0.0009), and IL-18 (*p* = 0.0057) were significantly decreased in the siCD74 + rGdMIF group compared to the sicontrol + rGdMIF group (Fig 6B–F). These data indicate that rGdMIF modulates inflammatory cytokine secretion through its interaction with CD74.

### 2.5. GdMIF is involved in *G. duodenalis* infection-caused intestinal inflammation and damage through NLRP3 pathway

**Fig 7:**
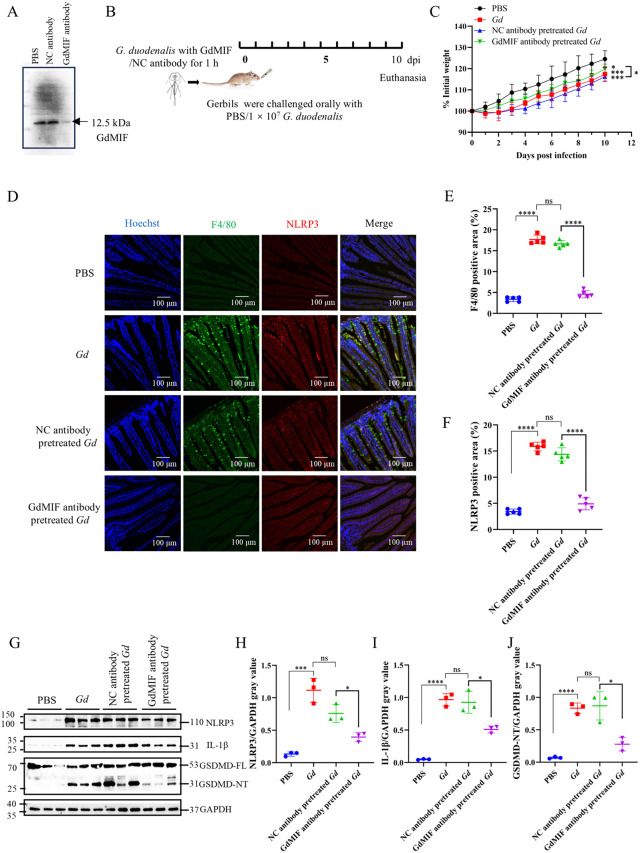
GdMIF induces NLRP3-GSDMD pathway activation in gerbil duodenum. (A) The ESPs of 1 × 10^7^ trophozoites incubated with PBS, NC serum (the serum of unimmunized mice, NC antibody) or anti-GdMIF polyclonal serum (GdMIF antibody) for 1 h were collected, and the secretion level of GdMIF in ESPs was detected by western blot. GdMIF antibody was utilized to detect GdMIF. (B) 1 × 10^7^ trophozoites were incubated with PBS, NC antibody or GdMIF antibody for 1 h, respectively, then trophozoites were used to infect gerbils, all gerbils were euthanized and collected duodenum at 10 dpi (n = 5 per group). (C) The body weights of gerbils were monitored for 10 dpi (n = 5 per group). (D) F4/80 and NLRP3 expression in duodenum was observed by IFA. Nuclei (blue), F4/80 (green) and NLRP3 (red), scale bar: 100 μm. Four images were analyzed in total per sample (n = 5). (E. F) The positive area of F4/80 and NLRP3 in duodenum image of per sample (n = 5) was quantified using Imag J. (G) The expression levels of NLRP3-GSDMD pathway related proteins in duodenum were detected by western blot (n = 3), and (H-J) The densitrometry analysis of NLRP3, IL-1β and GSDMD-NT protein. All results were representative of three number of biologically independent experiments. Data was shown as the mean ± SD, statistical significance was assessed using unpaired Student’s t-test. n.s. *p* > 0.05 indicates not significant, *p < 0.05, **p < 0.01, ***p < 0.001 and ****p < 0.0001.

**Fig 8:**
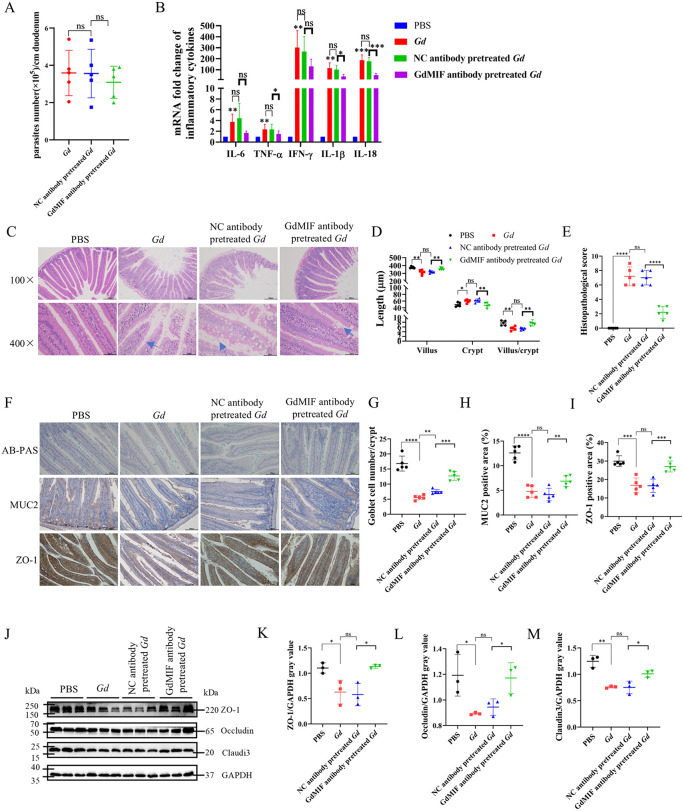
GdMIF secretion is involved in intestinal inflammation and injury caused by *G. duodenalis.* 1 × 10^7^ trophozoites were incubated with PBS, NC antibody or GdMIF antibody for 1 h, respectively, then trophozoites were used to infect gerbils, all gerbils were euthanized and collected duodenum at 10 dpi (n = 5 per group). (A) The number of trophozoites in the duodenal lavage fluid were counted. (B) The mRNA expression levels of cytokines in the duodenum were detected by qPCR. (C) Representative images of H&E staining in the duodenum. The blue arrow points to *G. duodenalis*, scale bar: 50 μm and 200 μm. Four images were captured in total per sample (n = 5), and the villus length and crypt depth were measured as indicated in the image. (D) The statistical analysis of villus length, crypt depth, and the ratio of the villus to crypt of the duodenum. (E) Histopathological score of duodenum. (F) AB-PAS staining of goblet cells in duodenum tissue, MUC2 and ZO-1 expression in duodenum tissue was observed via IHC, scale bar: 100 μm. Four images were captured in total per sample (n = 5). (G) The number of goblet cells in the image was analysed using Imag J (n = 5). (H) The level of MUC2 in the image was quantified using Imag J. (I) The level of ZO-1 in the image was quantified using Imag J. (J-M) The expression levels of ZO-1, Occludin, and Claudin3 proteins in duodenum were detected by western blot and densitometric analysis (n = 3). All results were representative of three number of biologically independent experiments. Data was shown as the mean ± SD, statistical significance was assessed using unpaired Student’s t-test. n.s. *p* > 0.05 indicates not significant, *p < 0.05, **p < 0.01, ***p < 0.001 and ****p < 0.0001.

To explore the role of GdMIF in *G. duodenalis* infection, the effect of using a GdMIF blocking antibody was tested *in vivo.* As shown in Fig 7A, GdMIF antibody pretreated *G. duodenalis* could block GdMIF secretion by *G. duodenalis*. Subsequently, gerbils were infected with *G. duodenalis* (Fig 7B), results showed that body weights of gerbils infected with *G. duodenalis*, the NC antibody pretreated *G. duodenalis* and the GdMIF antibody pretreated *G. duodenalis* were decreased compared with the PBS group. No significant difference in body weight was observed between the *Gd* group and the negative control (NC) antibody pretreated *Gd* group, but the body weight of the GdMIF antibody pretreated *Gd* group was increased compared with the NC antibody pretreated *Gd* group (Fig 7C). The number of *G. duodenalis* in gerbils duodenum in the GdMIF antibody pretreated *Gd* group was slightly less than that in the NC antibody pretreated *Gd* group, but there was no statistically significant difference (*p*> 0.5) (Fig 8A).

IFA result showed that enhanced macrophage recruitment and elevated NLRP3 expression, and NLRP3 in macrophage in the duodenum of both the *Gd* and the NC antibody-pretreated *Gd* groups compared to the PBS group (Fig 7D–F)*.* Western blot analysis further demonstrated that NLRP3, IL-1β, and GSDMD-NT protein were significantly increased in the *Gd* group and the NC antibody pretreated *Gd* group compared to the PBS group (Fig 7G–J)*.* Blocking the secretion of GdMIF significantly reduced macrophage recruitment, and the upregulation of NLRP3, IL-1β, and GSDMD-NT proteins compared to the NC antibody pretreated *Gd* group (Fig 7D–J). These results suggest that GdMIF triggers macrophage aggregation and activates the NLRP3 inflammasome in the duodenum.

The levels of IL-6, IFN-γ, IL-1β, and IL-18 mRNA were up-regulated in the *Gd* group and the NC antibody pretreated *Gd* group compared with the PBS group (Fig 8B), and IL-1β (*p* = 0.0279) and IL-18 (*p* = 0.0005) mRNA level was significantly decreased in the GdMIF antibody pretreated *Gd* group compared with the NC antibody pretreated *Gd* group, and IL-6 (*p* = 0.0941) and IFN-γ (*p* = 0.1268) mRNA levels were also decreased in the GdMIF antibody pretreated *Gd* group compared with the NC antibody pretreated *Gd* group, but no significant difference (Fig 8B). These results suggest that GdMIF is involved in *G. duodenalis*-caused inflammation in the duodenum.

HE staining of duodenal tissue revealed incomplete villi structures and extensive damage, crypt hyperplasia, severe pathological damage in both the *Gd* group and the NC antibody pretreated *Gd* group compared with the PBS group, while the duodenal villi were less damaged, crypt was normal, and pathological damage was weakened in the GdMIF antibody pretreated *Gd* group compared to the NC antibody pretreated *Gd* group (Fig 8C–E). To determine the effect of GdMIF on intestinal damage, goblet cells, MUC2, ZO-1, Occludin, and Claudin3 were examined. The study found that *G. duodenalis* infection led to significant reduction in MUC2, ZO-1, Occludin, and Claudin3 expression levels, as well as a decrease in goblet cell number in the duodenal tissue (Fig 8F–M). The *Gd* group and the NC antibody pretreated *G. d* group had about 66.9% (*p* < 0.0001) and 55.4% (*p* < 0.0001) fewer goblet cells than the PBS group, and the GdMIF antibody pretreated *Gd* group had about 24.4% (*p* = 0.0018) fewer goblet cells than the PBS group (Fig 8F and 8G). The expression amounts of MUC2 (Fig 8F and 8H) and ZO-1 (Fig 8F and 8I) in the GdMIF antibody pretreated *Gd* group were 0.65 (*p* = 0.0065) and 0.63 times (*p* = 0.001) higher than those in the NC antibody pretreated *Gd* group, respectively. These results suggest that GdMIF is involved in *G. duodenalis* infection-caused intestinal damage.

**Fig 9:**
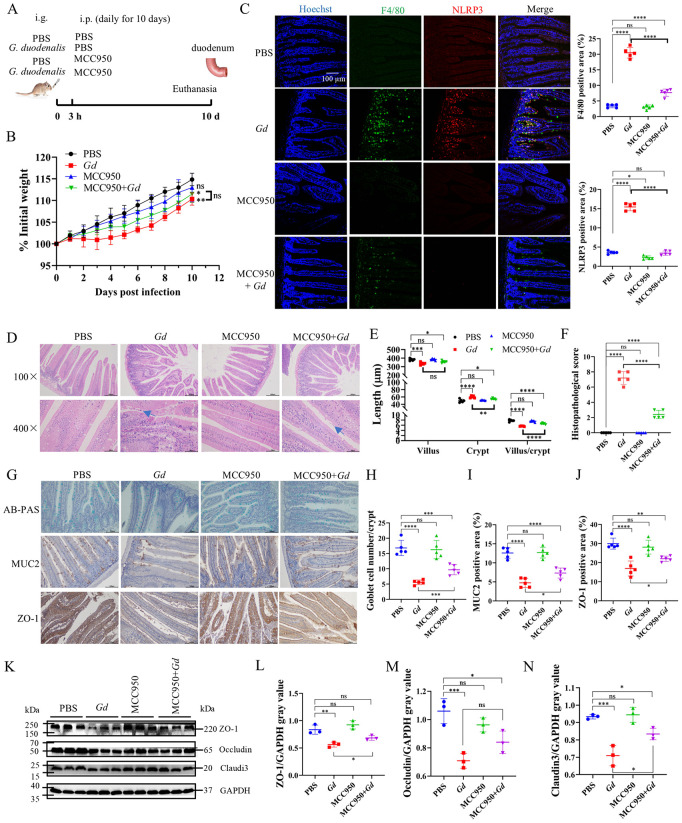
Inhibiting NLRP3 alleviates *G. duodenalis* caused intestinal injury. (A) Gerbils were administrated 1 × 10^7^ trophozoites or PBS for 3h, then gerbils were received MCC950 (10 mg/kg) i.p. daily for 10 days in the MCC950 inhibitor group. (B) The body weights of gerbils were monitored for 10 dpi (n = 5 per group). (C) F4/80 and NLRP3 expression in duodenum were observed by IFA. The nuclei were stained with hoechst (blue), F4/80 (green) and NLRP3 (red). Scale bar: 100 μm. Four images were analyzed in total per sample (n = 5). The positive area of F4/80 and NLRP3 in duodenum image of per sample (n = 5) was quantified using Imag J. (D) Representative images of H&E staining in the duodenum. The blue arrow points to *G. duodenalis*, scale bar: 50 μm and 200 μm. Four images were captured in total per sample (n = 5), and the villus length and crypt depth were measured as indicated in the image. (E) The statistical analysis of villus length, crypt depth, and the ratio of the villus to crypt of the duodenum. (F) Histopathological score of duodenum. (G) AB-PAS staining of goblet cells in duodenum tissue, MUC2 and ZO-1 expression in duodenum tissue was observed via IHC, scale bar: 100 μm. Four images were captured in total per sample (n = 5). (H) The number of goblet cells in the image was analysed using Imag J (n = 5). (I) The level of MUC2 in the image was quantified using Imag J. (J) The level of ZO-1 in the image was quantified using Imag J. (K-N) The expression levels of ZO-1, Occludin, and Claudin3 proteins in duodenum were detected by western blot and densitometric analysis (n = 3). All results were representative of three number of biologically independent experiments. Data was shown as the mean ± SD, statistical significance was assessed using using one-way ANOVA and unpaired Student’s t-test. n.s. *p* > 0.05 indicates not significant, *p < 0.05, **p < 0.01, ***p < 0.001 and ****p < 0.0001.

To further determine whether GdMIF regulated intestinal damage through NLRP3, NLRP3 inhibitor MCC950 was used in the gerbils (Fig 9A). The study showed that MCC950 treatment partially alleviated the weight loss induced by *G. duodenalis* infection (Fig 9B), inhibited *G. duodenalis* infection caused macrophage recruitment and NLRP3 expression in the duodenum (Fig 9C), and attenuated infection-induced villus blunting, crypt hyperplasia, and overall intestinal pathology scores (Fig 9D–F). Subsequently, we further examined the indicators related to the intestinal barrier, and the results showed the number of goblet cells in the MCC950 + *Gd* group was 1.74 times as much as that in the *Gd* group (*p* = 0.001) (Fig 9G and 9H). MUC2 and ZO-1 protein levels in the MCC950 + *Gd* group were 0.54 (*p* = 0.0122) and 0.3 times (*p* = 0.0253) higher than those in the *Gd* group, respectively (Fig 9G, 9I and 9J). Western blot analysis also confirmed that the expression levels of ZO-1, Occludin, and Claudin3 were elevated in the MCC950 + *Gd* group relative to the *Gd* group (Fig 9K–N). These results suggest that GdMIF is involved in *G. duodenalis* infection-caused intestinal damage through NLRP3 pathway.

## 3. Discussion

Recent studies have found that MIF homologues from *T. gondii*, *T. vaginalis*, *Plasmodium*, *Leishmania* are predicted to be structurally similar to HuMIF protein [27–30]. The amino acid sequences of parasite MIFs show significant homology with HuMIF, each consisting of around 115 residues and featuring identical conserved regions, including the N-terminal proline (Pro1), C-terminal cysteine (Cys60), and the tautomerase active site [14,36]. GdMIF gene with a HuMIF-like structure has been found in *G. duodenalis* [35]. The tautomerase active site is conserved thus both parasite and human MIF have tautomerase activity. To verify whether we purified rGdMIF retained enzymatic activity analogous to the native GdMIF protein, we assessed its dopamine tautomerase activity. We confirmed rGdMIF had dopamine tautomerase activity. Furthermore, studies have shown that HuMIF is localized in the cytoplasm, while the subcellular localization of parasite MIF varies across species. For example, *Plasmodium* MIF is predominantly expressed in the parasite cytoplasm [36]. *Leishmania* MIF is primarily localized to secretory vesicles within the parasite and may be released into host cells via exosomes or secretory pathways [37]. *G. duodenalis* has different morphological structures compared with other protozoa, we found that GdMIF was predominantly accumulated in the axial column and flagella in trophozoites. GdMIF was detected in GdESP.

There is a close functional link between MIF and NLRP3 inflammasome. HuMIF causes neuroinflammatory microglia pyroptosis by activating the NLRP3 inflammasome. Furthermore, the mouse MIF induces NLRP3 pathway activation in the kidney and liver [13]. TgMIF activates the NLRP3 inflammasome and causes hepatocyte pyroptosis [32]. *G. duodenalis* and its EVs have been reported to regulate TLR2 signaling pathway and active NLRP3 inflammasome [38,39]. *G. duodenalis* PPIB activates NLRP3 inflammasome and pyroptosis via TLR4 [40]. This study demonstrates that GdMIF modulates NLRP3 inflammasome activation and pyroptosis in mouse PMϕs.

CD74 is the primary receptor of MIF [41]. The ability of anti-CD74 antibodies to inhibit the phosphorylation of p44/p42 MAPK and neutrophil aggregation in MIF-induced macrophages [5]. In addition, Neutralizing CD74 suppresses MIF-induced fibroblasts proliferation [20]. Furthermore, it has been found that *Plasmodium* MIF and *E. histolytica* MIF can also bind to CD74 in host cells [34,42]. Whether GdMIF interacts with CD74 protein remains unclear. This study identifies an interaction between GdMIF and mouse CD74.

MIF regulates the NLRP3-GSDMD pathway by binding to CD74, thereby inducing lipid peroxidation, GSH depletion and ferroptosis in Type 2 diabetes mellitus [43]. Although accumulating evidence indicates that MIF is crucial for the NLRP3 inflammasome activation [44], there are no reports that MIF regulated NLRP3 inflammasome activation through interaction with host CD74. Therefore, we investigated whether GdMIF was involved in the *G. duodenalis* infection-induced NLRP3 activation by interacting with CD74. Our findings found that *G. duodenalis* upregulated CD74 protein expression in mouse PMϕs, and the interaction between GdMIF and CD74 was investigated through molecular docking and experimental verification. Knocking down CD74 receptor inhibited rGdMIF-induced NLRP3 inflammasome activation and pyroptosis pathways, reduced the release of IL-6, TNF-α, IFN-γ and IL-1β. NF-κB signal is the first signal of classical NLRP3 inflammasome activation [45,46]. Our data has revealed that rGdMIF induces the activation of the NF-κB signal, and the proteins of CD74, NF-κB and NLRP3-pyroptosis pathways show a significant positive correlation, indicating that there is a previous connection among them. Interestingly, we found that knocking down the expression of CD74 significantly inhibits the NF-κB pathway, and inhibiting NF-κB pathway could significantly reduce rGdMIF-induced NLRP3 inflammasome activation and pyroptosis. The study demonstrates that GdMIF activates the NF-κB-NLRP3 inflammasome and induces pyroptosis by interacting with mouse CD74, resulting in the secretion of pro-inflammatory cytokines in mouse PMϕs. As for the intestinal tract, we discovered that the activation of the NLRP3 inflammasome is related to macrophages through the F4/80 protein, it is also possible that cells in the body rely on this mechanism.

To further enhance the relevance of this research in the field of public health and to explore the potential for translation from mouse and gerbil models to human applications, we additionally conducted a computer simulation molecular docking analysis of the interaction between GdMIF and the human CD74 receptor. The results showed that there was also a strong binding ability between GdMIF and the human CD74 receptor, suggesting that this pathogenic mechanism may also exist in human cells. This discovery is of great significance, indicating that intervention strategies targeting the GdMIF-CD74 signaling pathway are not only applicable to experimental animal models but may also be used in the treatment of human giardiasis. This finding deepens our understanding of the complex interaction between MIF and host CD74.

Notably, MIF-induced the NLRP3 inflammatory response is involved in a variety of diseases [47,48]. Mouse MIF contributes to NLRP3 inflammasome-mediated pyroptosis in sepsis induced acute kidney injury [46]. While parasitic MIFs exhibit diverse and context-dependent functions. *P. yoelii* MIF can up-regulate the secretion of IFN-γ, TNF-α and IL-6 in mice, while also recruiting and activating monocytes [29], and *Leishmania* MIF modulates monocyte migration to evade immune responses. Notably, TgMIF activates the NLRP3 inflammasome, inducing hepatocyte pyroptosis to restrict parasite replication at the cost of severe liver damage [32]. In the case of *G. duodenalis*, while NLRP3 activation has been observed [40], its specific role and regulation remained unclear. In our study, we found that blocking GdMIF secretion significantly reduced the secretion of IL-6, IFN-γ, IL-1β, and IL-18, and the recruitment of macrophages in the gerbil duodenum. From this, we speculated that the activation of NLRP3 inflammasome and pyroptosis in macrophages induced by GdMIF maid play an important role in giardiasis. Our study found that blocking GdMIF secretion by *G. duodenalis* had little effect on parasite loads, but *G. duodenalis* infection-mediated NLRP3 inflammasome activation and pyroptosis, and duodenal inflammation and damage relieved after blocking GdMIF. Crucially, this protective effect was not attributable to parasite clearance, as parasite burdens remained unchanged, highlighting a direct pathophysiological role of GdMIF in provoking host-mediated inflammatory damage. This mechanism stands in stark contrast to the host-protective role of TgMIF-induced pyroptosis, underscoring the divergent evolutionary strategies employed by different parasites. The therapeutic potential of targeting this pathway was further confirmed by the fact that the NLRP3 inhibitor MCC950 similarly alleviated duodenal injury. Our findings position GdMIF-induced NLRP3 activation as a critical mediator of giardiasis pathology.

**Fig 10:**
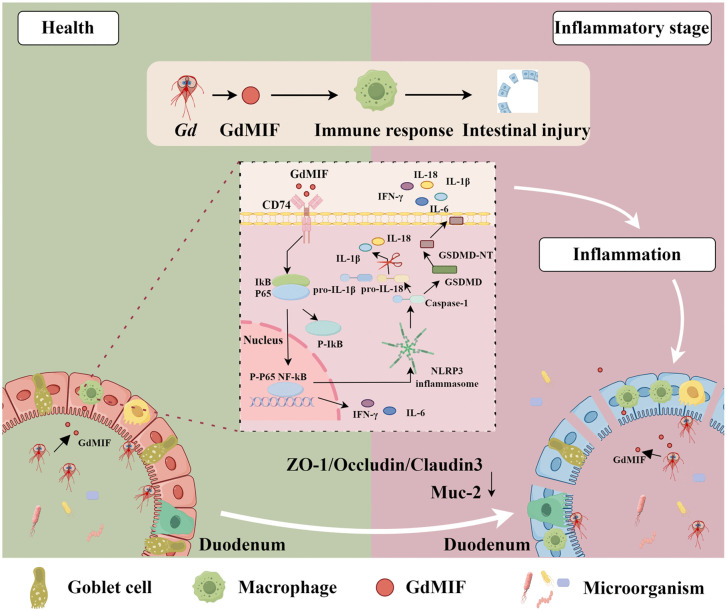
Schematic of the role mechanism of GdMIF in *G. duodenalis* infection. GdMIF induced NF-κB-NLRP3 inflammasome activation and pyroptosis by binding to CD74, leading to pro-inflammatory cytokines secretion, which was involved in *G. duodenalis-*caused intestinal damage.

In summary, our findings indicate that GdMIF induces NF-κB-NLRP3 inflammasome activation and pyroptosis by binding to CD74, leading to pro-inflammatory cytokines secretion, which is involved in *G. duodenalis-*caused intestinal damage (Fig 10). This identifies a potential target for giardiasis prevention and treatment. Future research can focus on developing small molecule inhibitors or antibody drugs that specifically block the binding of GdMIF to human CD74, thereby providing new treatment methods for controlling giardiasis and related intestinal inflammatory damage. At the same time, this mechanism also helps to deepen the understanding of the pathogenesis of other parasitic diseases and inflammatory-related diseases, and has broad potential medical value.

## 4. Materials and methods

### 4.1. Ethics statement

All gerbil experiments were approved by the Animal Welfare and Research Ethics Committee of Jilin University (IACUC License No. SY202007001).

### 4.2. Animal

Specific Pathogen-Free (SPF) Mongolian gerbils were a gift from Professor Yuanhua Qin of Dalian Medical University. Gerbils were housed in a specific pathogen-free environment, following a 12 h light/dark cycle, at a controlled temperature (22 ± 2°C), and maintained at a relative humidity of 50 ± 5%.

### 4.3. *G. duodenalis* preparation

*G. duodenalis* trophozoites (WB strain, ATCC30957) were cultured in modified TYI-S-33 medium with 10% fetal bovine serum (FBS, Cat. No. 13011–8611, Every Green), 0.1% bovine bile (Cat. No. B8381, Sigma-Aldrich) and 1% Penicillin-Streptomycin solution (Cat. No. P0781, Sigma-Aldrich) at 37°C under microaerophilic conditions.

### 4.4. Protein expression and purification

*GdMIF* gene (GL50803_0012091) was amplified by PCR using primers (S1 Table) and *G. duodenalis* cDNA as templates. The PCR products were inserted into the pET-32a-His vector. The recombinant plasmids of pET32a-*GdMIF* were transformed into *Escherichia coli* BL21 (DE3). For rGdMIF expression, the transformed bacteria (pET32a-*GdMIF*) was cultured and induced with 1 mM isopropyl-β-D-thiogalactopyranoside (IPTG) (Cat. No. BS119, Biosharp) at 37°C with shaking. Then, the bacterial solution was ultrasonically broken by ultrasonic treatment and centrifuged at 8,000 × *g* for 5 min to collect the precipitation and supernatant. The rGdMIF and the total bacterial protein from IPTG-induced DE3 harboring the pET32a (+) empty vector were purified using a Ni-NTA column (Cat. No. HY-K0220, Beyotime). To remove endotoxin, rGdMIF and His-tag protein were treated with 1% Triton X-114 (v/v) (Cat. No. S15023, YuanYe Biotechnology) at 4°C for 30 min, followed by phase separation at 37°C for 10 min and centrifugation at 12,000 × *g* for 10 min to collect the aqueous phase. This procedure was repeated three to five times. Subsequently, residual Triton X-114 was effectively eliminated from the protein solution by extensive dialysis against PBS using a membrane with a molecular weight cutoff of 10 kDa [49]. Endotoxin levels were quantified using the Limulus Amebocyte Lysate (LAL) assay (Cat. No. L00350C, GenScript), and the endotoxin content of rGdMIF is 0.068 EU/mL and the endotoxin content of His-tag protein is 0.097 EU/mL.

### 4.5. Dopachrome tautomerase activity examination

Given that dopachrome tautomerase activity is a hallmark feature of MIF proteins with a highly conserved active site, we assessed this activity to verify we expressed the protein was rGdMIF and that it had biological activity. The dopachrome tautomerase activity of rGdMIF was assessed according to an established method. Briefly, the substrate was freshly prepared by mixing 8 mM sodium periodate with 4 mM L-3,4-dihydroxyphenylalanine methyl ester (Cat. No. 28900-64-3, YuanYe Biotechnology) for 10 min, followed by incubation on ice for an additional 10 min. The enzymatic reaction was initiated by adding the prepared substrate into a cuvette containing 200 nM rGdMIF in 25 mM potassium phosphate buffer supplemented with 0.5 mM EDTA. The reaction kinetics were monitored by measuring the decrease in absorbance at 475 nm over a 15 min period using a fluorescent microplate reader [44].

### 4.6. Preparation of anti-rGdMIF polyclonal serum

The purified rGdMIF protein (100 μg/mouse) was emulsified with an equal volume of Freund’s complete adjuvant for the primary subcutaneous immunization of BALB/C mice. Booster immunizations were administered at two-week intervals using the same amount of antigen emulsified in Freund’s incomplete adjuvant. A test bleed was performed 10 days after the third immunization to monitor the immune response. The antibody titer and specificity against rGdMIF were assessed by enzyme-linked immunosorbent assay (ELISA) and western blot, respectively. Upon confirmation of a high antiserum titer, a terminal bleed was conducted. Serum was separated by incubating the clotted blood at 4°C overnight, followed by centrifugation at 3,000 × *g* for 15 min, the final serum antibody was stored at -80°C.

### 4.7. Localization of GdMIF by immunofluorescence assays (IFA)

*G. duodenalis* trophozoites (5 × 10^5^) were adhered to a glass slide pretreated with polylysine in a 24-well plate, and the trophozoites were fixed with 4% paraformaldehyde at room temperature (RT) for 20 min, then permeabilized with 0.5% Triton X-100 at RT for 20 min, they were then blocked with 3% BSA at RT for 30 min. Subsequently, the trophozoites were incubated with mouse anti-GdMIF polyclonal serum (GdMIF antibody, 1:200) at 4°C overnight. Following three washes, the trophozoites were incubated with Alexa Fluor 488-conjugated Affinipure Goat Anti-Mouse IgG (Cat. No. SA00013–1, Proteintech) for 1 h at 37°C, shielded from light. After washing, the slides were sealed with Hoechst 33342 (Cat. No. P0133, Beyotime) containing an anti-fluorescence quench agent. Finally, the samples were observed by FV3000 (Olympus, Tokyo, Japan). Five fields of view were analyzed in total per experiment, and three biologically independent experiments were performed.

### 4.8. Detection of secreted GdMIF in *G. duodenalis* excretory-secretory products (GdESP)

The *G. duodenalis* trophozoites were resuspended at a concentration of 1 × 10⁶ parasites/mL in modified TYI-S-33 medium supplemented with depleted fetal bovine serum and maintained under standard culture conditions (37°C, 12 h), then cultured using 1640 medium (Cat. No. R2405, Sigma-Aldrich) for 6 h. Cell-free supernatants were obtained through sequential centrifugation steps: initial clarification at 2,000 × *g* for 10 min (4°C), followed by high-speed centrifugation at 10,000 × *g* for 45 min (4°C) [50]. The final supernatant fractions were collected and concentrated by the methanol-chloroform method for subsequent analysis. Polyvinylidene fluoride (PVDF) membranes (Millipore, USA) were used to transfer GdESP protein after SDS-PAGE. The anti-GdMIF mouse serum (1:1,000) incubated the PVDF membrane overnight at 4°C, and then HRP anti-mouse IgG (Cat. No. SA00001–1, Proteintech) incubated PVDF membrane for 1 h. The membrane was then treated with a chemiluminescent substrate and visualized using the Clinx ChemiScope Series (Clinx, Shanghai, China).

### 4.9. Isolation and cultivation of mouse peritoneal macrophage (PMϕs)

Mouse was intraperitoneally injected with 2 mL sterile 2.98% Difo Fluid Thioglycolate Medium (Cat. No. BD-226382, BD) and euthanized at 3 d post-injection. The peritoneal cavity was rinsed with 3 mL cold PBS. The collected PMϕs suspension was centrifuged at 1,000 × *g* for 10 min, and PMϕs were plated in cell culture plates and cultured in 1640 medium with 10% FBS (Cat. No. 9014-81-7, Sigma-Aldrich) and 1% Penicillin-Streptomycin solution (Cat. No. P0781, Sigma-Aldrich) at 37°C with 5% CO_2_ for 6 h [51].

### 4.10. Real-time quantitative PCR (qPCR)

PMϕs were incubated with 0.1 μg/mL, 0.5 μg/mL, 1 μg/mL rGdMIF for 24 h, the control group was treated with an equal volume of sterile PBS, *G. duodenalis* were washed and resuspended in sterile PBS immediately prior to co-incubation with PMϕs (MOI = cell:parasite = 3) as the positive control group. 1 × 10^7^ trophozoites were incubated with PBS, NC antibody or GdMIF antibody for 1 h, respectively, then trophozoites were used to infect gerbils. All gerbils were euthanized, and the duodenum was collected at 10 days. PMϕs and duodenum RNA were isolated using TRIzol reagent (Cat. No. YY101L, Epizyme) and converted to cDNA with the PrimeScript RT reagent Kit (Cat. No. RR037Q, Takara). Gene transcription levels were measured using qPCR assays on a LightCycler 480 II platform (Roche Diagnostics GmbH, Mannheim, Germany) with 2 × Universal SYBR Green qPCR Master Mix (Cat. No. G3326, Servicebio). Data were normalized to GAPDH, and relative mRNA fold-changes were determined using the 2^−ΔΔCq^ method. Primers (S1 Table) were designed with the PrimerQuest Tool (https://sg.idtdna.com/pages/) and synthesized by Kumei Biotechnology Co., Ltd (Changchun, China).

### 4.11. Western blot analysis

PMϕs were incubated with 1 μg/mL His, 1 μg/mL rGdMIF, or *G. duodenalis* (MOI = 3) for 24 h, or were incubated with 1 μg/mL rGdMIF or His for 6, 12, 24 h, respectively. PMϕs were transfected with control-siRNA (sicontrol) and CD74-siRNA (siCD74) using Lipo2000 and cultured for 24 h, and then stimulated with rGdMIF (1 μg/mL) for 24 h; PMϕs were pretreated with BAY11–7082 (5 μM, Cat. No. HY-13453, MCE) for 2 h and then stimulated with rGdMIF (1 μg/mL) for 24 h. PMϕs or duodenum were lysed using RIPA Lysis Buffer (Cat. No. P0013J, Beyotime). Each sample’s 30 µg protein was separated using SDS-PAGE and transferred to a PVDF membrane. Membranes were blocked with a protein-free rapid blocking solution (Cat. No. PS108, EpiZyme) for 30 min, followed by overnight incubation at 4°C with antibodies against NLRP3 (Cat. No. AG-20B-0014-C100, Adipogen), IL-1β (Cat. No. xy-2022, R&D), caspase-1 (p20) (Cat. No. AG-20B-0042-C100, Adipogen), GSDMD (Cat. No. 36425T, Cell Signaling Technology), CD74 (Cat. No. YP-Ab-14028, Youbao), P-p65 (Cat. No. 3033T, Cell Signaling Technology), NF-κB p65 (Cat. No. 8242T, Cell Signaling Technology), P-IκBα (Cat. No. 2859T, Cell Signaling Technology), IκBα (Cat. No. 4812T, Cell Signaling Technology), ZO-1 (Cat. No. AF5145, Affinity), Occludin (Cat. No. db11580, Diagbio), Claudin3 (Cat. No. db13582, Diagbio), and GAPDH (Cat. No. 60004–1-Ig, Proteintech). The membranes were incubated with HRP anti-rabbit IgG antibody (Cat. No. SA00001–2, Proteintech), HRP anti-mouse IgG antibody, or HRP-conjugated rabbit anti-goat IgG antibody (Cat. No. SA00001–4, Proteintech) at RT for 1 h. The membrane was detected with enhanced chemiluminescence solution from EpiZyme (Cat. No. SQ201, EpiZyme) and blot was visualized on a Clinx ChemiScope Series (Clinx, Shanghai, China), protein densitometry analysis was performed using Image J.

### 4.12. Immunofluorescence assay (IFA)

For detection of NLRP3 in PMϕs, PMϕs were plated on sterile slides in 24-well plates. PMϕs were incubated with 1 μg/mL His, 1 μg/mL rGdMIF, or *G. duodenalis* (MOI = 3) for 24 h; PMϕs were transfected with control-siRNA (sicontrol) and CD74-siRNA (siCD74) using Lipo2000 and cultured for 24 h, and then stimulated with rGdMIF (1 μg/mL) for 24 h; PMϕs were pretreated with BAY11–7082 (5 μM, Cat. No. HY-13453, MCE) for 2 h and then stimulated with rGdMIF (1 μg/mL) for 24 h. The cells were then fixed in 4% paraformaldehyde and permeabilized with 0.5% Triton X-100, both at RT for 20 min. Cells were blocked with 3% BSA at RT for 30 min, followed by overnight incubation with NLRP3 (1:200) or CD74 (1:200) primary antibody at 4°C. Cells were incubated with CoraLite594-conjugated Recombinant Rabbit Anti-Mouse IgG (Cat. No. SA00014–2, Proteintech) at RT for 1 h, followed by sealing the slides with Hoechst33342 containing an anti-fluorescence quench agent. Finally, three images of view were captured in total per sample by FV3000, quantitative analysis of the presence of NLPR3 signals in x% of the cells in each image was performed using Image J software, and three biologically independent experiments were performed.

For detection of NLRP3 and F4/80 in duodenum, after routine dewaxing, paraffin sections of the duodenum were boiled in EDTA/Tris buffer for 10 min. The tissue sections were rinsed 3 times with PBS. After blocking with 3% BSA for 2 h, the tissue sections were incubated with NLRP3 (1:200) and F4/80 (Cat. No. DF2789, Affinity) (1:200) antibodies at 4°C overnight. Following washes, sections were incubated with CoraLite594-conjugated Recombinant Rabbit Anti-Mouse IgG and CoraLite488-conjugated Recombinant Goat Anti-Rabbit IgG at RT for 45 min, then sealed with Hoechst33342 containing an anti-fluorescence quenching agent. Finally, the samples were observed by FV3000. Four fields of view were analyzed in total per sample, and three biologically independent experiments were performed.

### 4.13. Lactate dehydrogenase (LDH) release assay

The cell culture supernatants from various groups were collected and centrifuged at 12,000 × *g* for 5 min. The release of LDH in cellular supernatant was assessed using a commercial LDH cytotoxicity assay kit following the manufacturer’s guidelines (Cat. No. C0016, Beyotime). Absorbance was recorded at a wavelength of 490 nm.

### 4.14. Protein-protein interaction analysis

The protein structures of GdMIF (V6TQY6), mouse CD74 (P04441) and human CD74 (P04233) were searched in the UniProtKB database (https://www.uniprot.org), and the proteins’ 3D structure were analyzed by the AlphaFold Protein Structure Database (https://alphafold.com/). Protein-protein docking was performed using the GRAMM server with default parameters. The resulting complexes were rigorously evaluated, with the binding energy serving as the primary criterion, a threshold of <-4 kcal/mol was applied, where lower values indicate a more stable complex. The top-ranked docking pose was selected for in-depth analysis based on complementary metrics, including the buried surface area at the interface, hydrogen bonding interactions, and key amino acid residues involved in binding. Furthermore, the binding free energy was quantitatively calculated using the PDBePISA server. The final docking result was visualized by Pymol software (https://www.pymol.org/).

### 4.15. Co-immunoprecipitation (Co-IP)

*CD74* gene (NM_010545) was amplified by PCR using primers (S1 Table) and mouse PMϕs cDNA as templates. The GdMIF and CD74 PCR products were ligated into pcDNA3.1-His and pcDNA3.1-N-HA vectors, respectively. The pcDNA3.1-His-GdMIF plasmid and the pcDNA3.1-N-HA-CD74 plasmid were co-transfected into HEK293T cells. The cells were lysed by cell lysis buffer (Cat. No. P0013J, Beyotime) with 1 mM PMSF solution (Cat. No. ST505, Beyotime), and then the supernatants were collected, which were then incubated overnight at 4°C with shaking using either anti-HA (Cat. No. 51064–2-AP, Proteintech) or IgG antibodies (Cat. No. 30000–0-AP, Proteintech). The immune complex solution was mixed with protein A/G magnetic beads and incubated for 1 h at RT. After extensive washing, the bound proteins were eluted from the beads by boiling in 1 × SDS loading buffer for 10 minutes, then sample elutions were subsequently separated using a magnetic rack and subjected to western blot analysis.

### 4.16. Bimolecular fluorescence complementation assays (BiFC)

Target genes (*GdMIF* gene and mouse *CD74* gene) were connected with BiFC negative plasmid (pBiFC-VN173 and pBiFC-VC155) to construct recombinant plasmids pBiFC-GdMIF-VN173 and pBiFC-CD74-VC155. pBiFC-bJun-VN173 plasmid and pBiFC-bFos-VC155 plasmid (as positive control), pBiFC-VN173 plasmid and pBiFC-VC155 plasmid (as negative control), pBiFC-GdMIF-VN173 plasmid and pBiFC-CD74-VC155 plasmid were transfected into HEK293T cells via Lipofectamine 2000 (Cat. No. 11668–019, Invitrogen). The untreated HEK293T cell group was used as the Control group. Fluorescence was observed via FV3000. Three fields of view were analyzed in total per sample, and three biologically independent experiments were performed.

### 4.17. Enzyme-linked immunosorbent assays

Cell culture supernatants from each group were collected, and the levels of pro-inflammatory cytokines IL-6 (Cat. No. 88-7064-88, Invitrogen), IL-12 (Cat. No. 88-7120-22, Invitrogen), IL-1β (Cat. No. 88-7013A-88, Invitrogen), IL-18 (Cat. No. 88-50618-77, Invitrogen), and TNF-α (Cat. No. 88-7324-88, Invitrogen) were quantified using ELISA kits following the manufacturer’s guidelines. Absorbance was collected at 450 nm.

### 4.18. Animal model

To investigate the role of GdMIF in *G. duodenalis* infection *in vivo*, we first assessed the neutralizing efficacy of the anti-GdMIF antibody by measuring its impact on GdMIF secretion from trophozoites. 1 × 10^7^ trophozoites incubated with PBS, Normal control serum (the serum of unimmunized mice, NC antibody) or anti-GdMIF polyclonal serum (GdMIF antibody) for 1 h, respectively. After incubation, trophozoites were washed three times with sterile PBS to remove unbound antibodies and then cultured in RPMI-1640 medium (Cat. No. R2405, Sigma-Aldrich) for 6 h to collect GdESPs, then cell-free supernatants were obtained through clarification at 2,000 × *g* for 10 min (4°C). The proteins in GdESPs were concentrated into protein chunks, and then the protein chunks were dissolved in 1 × SDS and prepared as protein samples for western blot analysis using the GdMIF antibody for detection.

4 week old gerbils were randomly assigned to four groups (n = 5 per group), investigators were not involved in group allocation. (i) the PBS group: gerbil was administered with 100 μL sterile phosphate-buffered saline (PBS) via intragastric gavage (i.g.). (ii) the *Gd* group: gerbil was administered 1 × 10^7^ trophozoites that had been pre-incubated with PBS for 1 h suspended in 100 μL PBS. (iii) the negative control (NC) antibody pretreated *Gd* group: gerbil was administered 1 × 10^7^ tachyzoites that had been pre-incubated with NC antibody for 1 h suspended in 100 μL PBS. (iv) the GdMIF antibody pretreated *Gd* group: gerbil was administered 1 × 10^7^ tachyzoites that had been pre-incubated with GdMIF antibody for 1 h suspended in 100 μL PBS [27,38].

4 week old gerbils were divided into four groups (n = 5 per group): (i) the PBS group: 100 µL/gerbil PBS by gavage, followed 3 h later by 100 µL/gerbil PBS intraperitoneal injection (i.p.) route daily for 10 days. (ii) the *Gd* group: 1 × 10^7^ trophozoites/gerbil suspended in 100 μL PBS by gavage, followed 3 h later by 100 µL/gerbil PBS i.p. route daily for 10 days. (iii) the MCC950 (NLRP3 inhibitor, Cat. No. HY-12815, MCE) group: 100 µL/gerbil PBS by gavage, followed 3 h later by 10 mg/kg body weight MCC950 suspended in 100 μL PBS by i.p. route daily for 10 days. (iv) the MCC950 + *Gd* group: 1 × 10^7^ trophozoites/gerbil suspended in 100 μL PBS by gavage, followed 3 h later by 10 mg/kg body weight MCC950 suspended in 100 μL PBS by i.p. route daily for 10 days.

Gerbil body weight was monitored daily. At 10 days post-infection (dpi), gerbils were anesthetized with 3% isoflurane and euthanized by an intraperitoneal injection of sodium pentobarbital (150 mg/kg), followed by cervical dislocation. The duodenum was harvested, and a 2 cm segment was immersed in 1 mL of ice-cold PBS and kept on ice for 30 min to detach adherent trophozoites. The tissue was then rinsed ten times with fresh PBS. The resulting wash suspensions were pooled, and the number of detached trophozoites was quantified by counting under an Olympus CX23 optical microscope at 200 × magnification using a standard hemocytometer (Improved Neubauer chamber). Additionally, the duodenum was collected for analysis using western blot, qPCR, histological observation, AB-PAS, immunofluorescence, and immunohistochemical staining assays [39].

### 4.19. Histopathological observation

Duodenum samples were collected, fixed in 4% paraformaldehyde, embedded in paraffin, and sectioned into 5 μm-thick slices. Tissue sections were deparaffinized in xylene, rehydrated through a graded ethanol series, and stained with hematoxylin and eosin (H&E). Pathological changes were observed by two independent pathologists blinded to the experimental groups, and for each sample, four non-overlapping fields were systematically captured, villus length and crypt depth were measured using image analysis software, and three biologically independent experiments were performed. A semi-quantitative scoring system was employed to evaluate the severity of duodenal damage [52]. The following parameters were scored on a scale of 0–3. (i) inflammatory cell infiltration, (ii) tissue damage and crypt loss, (iii) villus architectural distortion, and (iv) goblet cell depletion. The scores for each parameter were summed to generate a total histopathology score ranging from 0 (normal) to 12 (severe damage) per field of view.

### 4.20. Alcian blue PAS (AB-PAS) staining

AB-PAS staining was used to label the goblet cells. Tissue sections were deparaffinized in xylene, rehydrated through a graded ethanol series, and stained using the AB-PAS stain kit (Cat. No. G1049, Servicebio) as per the manufacturer’s instructions. Sections were observed by two independent pathologists blinded to the experimental groups, and four non-overlapping fields were systematically captured for each sample, and three biologically independent experiments were performed. The number of goblet cells in crypts in the image was analyzed using Image J.

### 4.21. Immunohistochemical (IHC) staining

After routine dewaxing, the duodenum sections were boiled in citrate buffer for 10 min. The sections were treated with ZO-1 (Cat. No. AF5145, Affinity) and MUC2 (Cat. No. DF8390, Affinity) antibodies, goat-anti-rabbit IgG (HRP), and DAB staining (Cat. No. DA1010, Solarbio) following the UltraSensitive SP (mouse/rabbit) IHC Kit instructions (Cat. No. KIT-9710, Maixin Biotechnology). Sections were observed by two independent pathologists blinded to the experimental groups, and four non-overlapping fields were systematically captured for each sample, and three biologically independent experiments were performed, then the levels of MUC2 and ZO-1 in the image were quantified using Image J.

### 4.22. Statistical analysis

All values are expressed as the mean ± SD of three independent experiments. Prior to parametric testing, the normality of all data groups was confirmed with the Shapiro-Wilk test. Statistical analyses were performed using GraphPad Prism 6.01 (GraphPad, California, USA). The unpaired Student’s t-test was employed to compare data between two groups, while a one-way ANOVA was utilized for comparisons among multiple groups. The boxes and error bars in the figures indicate the mean value and standard deviation, respectively. Significance levels are indicated as follows: *p < 0.05, **p < 0.01, ***p < 0.001 and ****p < 0.0001.

## Supporting information

S1 Fig The specificity of GdMIF antibody. (A-B) The specificity of GdMIF antibody was detected by western blot. The GdESPs was collected and used to prepare protein sample. The protein sample was then subjected to SDS-PAGE and transferred onto a PVDF membrane. The PVDF membrane was incubated with GdMIF antibody (A) or NC antibody (B) to evaluate the specificity of the GdMIF antibody.
(TIF)

S2 Fig The computer simulation molecular docking analysis of the interaction between GdMIF and the human CD74 receptor. The molecular docking model of GdMIF (yellow) with human CD74 (blue) protein.
(TIF)

S1 Table Primers used in this study. (DOCX)

S1 Data Excel spreadsheet containing, in separate sheets, the underlying numerical data and statistical analysis for Figure panels in the main text. (XLSX)

S2 Data All the original western blot images in the study. (DOCX)
