# Supplementary figures and images for: Giardia duodenalis MIF induces host intestinal damage via CD74 receptor mediated NLRP3 inflammasome activation
Source: PLoS Negl Trop Dis. 2026 Feb 2;20(2):e0013968. doi: 10.1371/journal.pntd.0013968 (PMC12880751; doi:10.1371/journal.pntd.0013968)

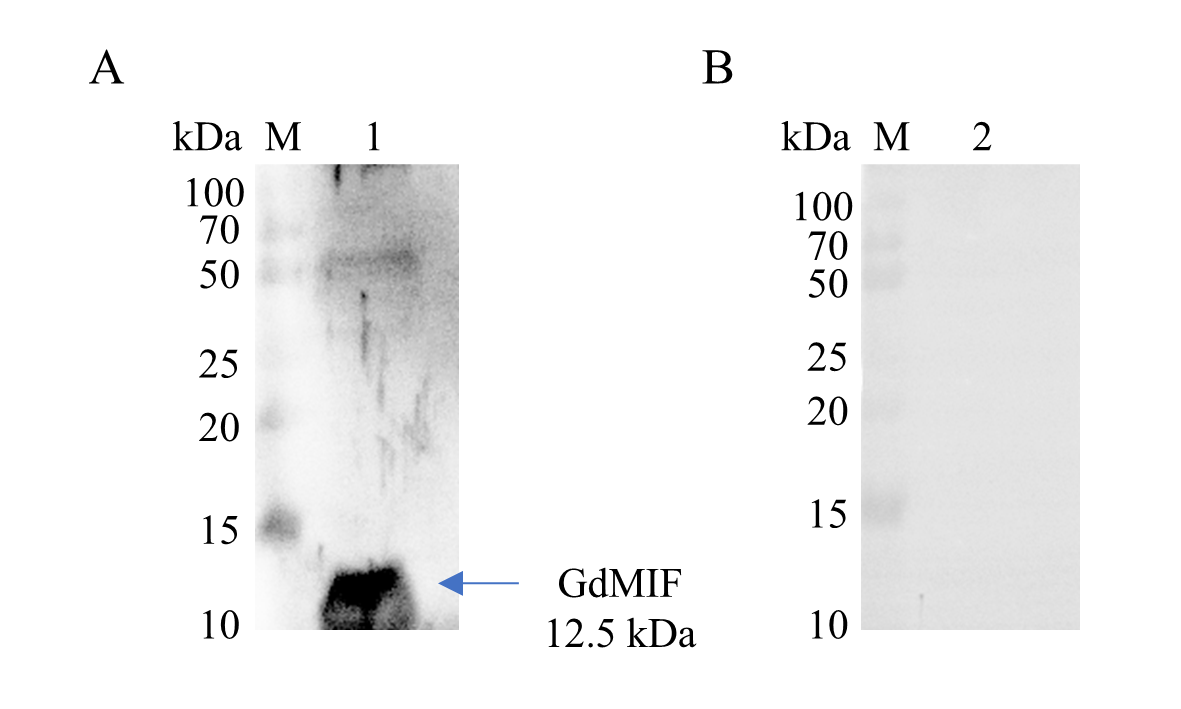

Supplement: S1 Fig — (A-B) The specificity of GdMIF antibody was detected by western blot. The GdESPs was collected and used to prepare protein sample. The protein sample was then subjected to SDS-PAGE and transferred onto a PVDF membrane. The PVDF membrane was incubated with GdMIF antibody (A) or NC antibody (B) to evaluate the specificity of the GdMIF antibody. (TIF) [file pntd.0013968.s001.tif]

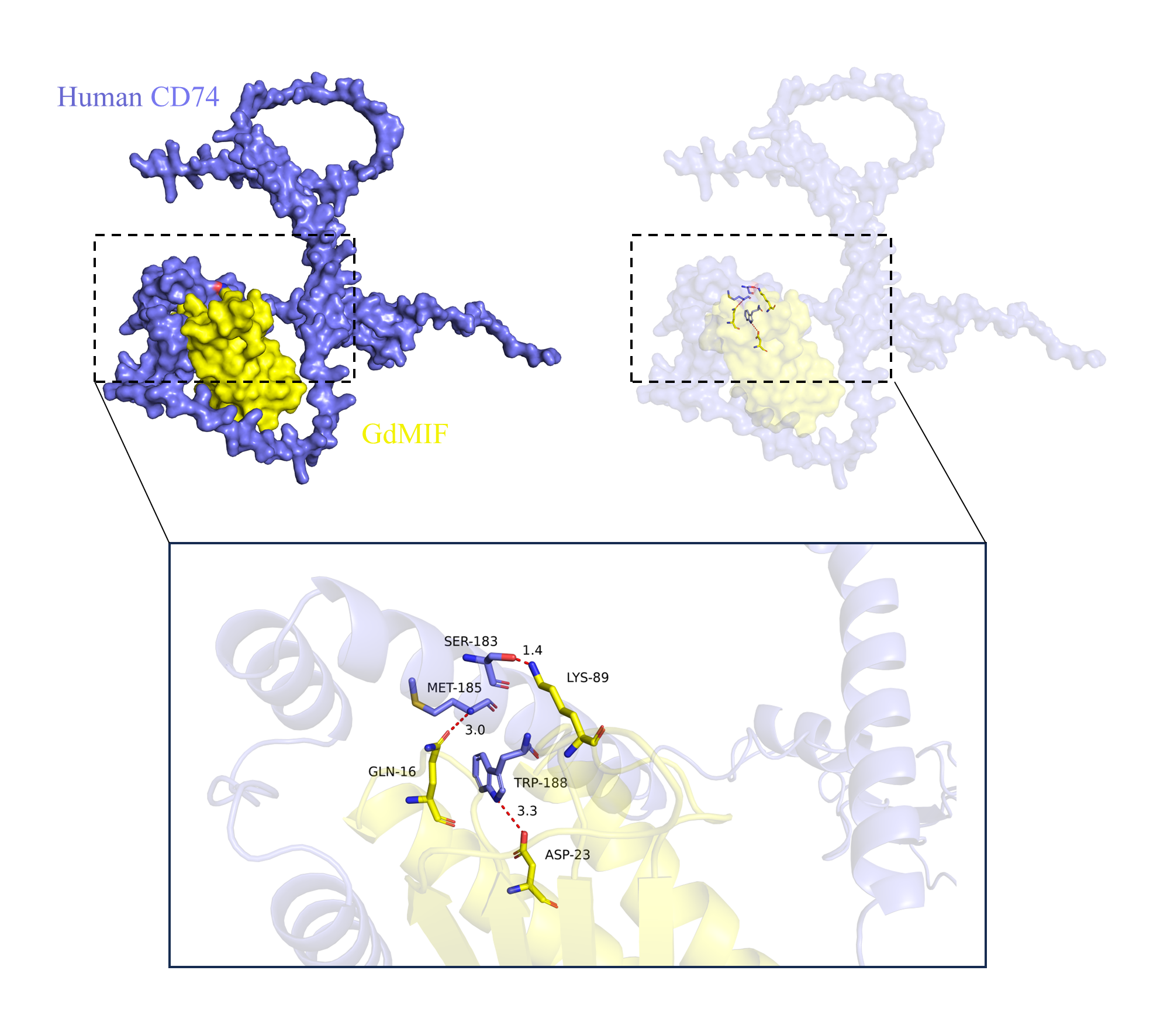

Supplement: S2 Fig — The molecular docking model of GdMIF (yellow) with human CD74 (blue) protein. (TIF) [file pntd.0013968.s002.tif]
