# Supplementary material for: Giardia duodenalis MIF induces host intestinal damage via CD74 receptor mediated NLRP3 inflammasome activation
Source: PLoS Negl Trop Dis. 2026 Feb 2;20(2):e0013968. doi: 10.1371/journal.pntd.0013968 (PMC12880751; doi:10.1371/journal.pntd.0013968)
Supplement: S1 Table — (DOCX) [file pntd.0013968.s003.docx]

| **S1 Table. Primers used in this study** | |
| --- | --- |
| **Primer** | **Primer Sequence (5’ to 3’)** |
| Primers for prokaryotic and eukaryotic expression | |
| pET-32a-GdMIF-F | GCCATGGCTGATATCGCTATGCCTTGCGCCATTGTC |
| pET-32a-GdMIF-R | GAGCTCGAATTCGGATCAAACGTGCTGCCATTAAAG |
| pcDNA3.1-His-GdMIF-F | CACCACACTGGACTAGTGATGCCTTGCGCCATTGT |
| pcDNA3.1-His-GdMIF-R | GGTACCGAGCTCGGATCGAAACGTGCTGCCATTAAAGCC |
| pcDNA3.1-N-HA-CD74-F | CTTGGTACCGAGCTCGACATGGATGACCAACGCGAC |
| pcDNA3.1-N-HA-CD74-R | ACACTGGACTAGTGGATCCAGGGTGACTTGACCCAG |
| pBiFC-GdMIF-VN173-F | GACAAGCTTGCGGCCGCCATGCCTTGCGCCATTG |
| pBiFC-GdMIF-VN173-R | CCGATATCAGATCTATCGAAAACGTGCTGCCATTAAAGCC |
| pBiFC-CD74-VC155-F | CTTATGGCCATGGAGGCCATGGATGACCAACGCGAC |
| pBiFC-CD74-VC155-R | AGAGATCTCGGTCGACCTCAGGGTGACTTGACCCAG |
| Primers for qPCR | |
| NOD1-F | GATTGGAGACGAAGGGGCAA |
| NOD1-R | CGTCTGGTTCACTCTCAGCA |
| NOD2-F | GCCAGTACGAGTGTGAGGAG |
| NOD2-R | GCGAGACTGAGTCAACACCA |
| NLRP1-F | ATAAACAAGCCACCCCCAGT |
| NLRP1-R | TGTGCCCAATGTCGATCTCA |
| NLRP3-F | AGCCAGAGTGGAATGACACG |
| NLRP3-R | CGTGTAGCGACTGTTGAGGT |
| NLRP6-F | TTGTTCGACAGGCTCTCAGC |
| NLRP6-R | ACTGGGGGTTGTTTCTTGGT |
| NLRC4-F | GCTCAGTCCTCAGAACCTGC |
| NLRC4-R | ACCCAAGCTGTCAATCAGACC |
| NLRC5-F | TCTCTAAGCAGCTAGGGGCA |
| NLRC5-R | GGGGAGTGAGGAGTAAGCCA |
| Mus-IL-6-F | TGCCTTCTTGGGACTGATGC |
| Mus-IL-6-R | GCAAGTGCATCATCGTTGTTC |
| Mus-IL-10-F | GCAGTGGAGCAGGTGAAGAG |
| Mus-IL-10-R | CGGAGAGAGGTACAAACGAGG |
| Mus-IL-12-F | TACAAGGTTCAGGTGCGAGC |
| Mus-IL-12-R | ATGTATCCGAGACTGCCCAC |
| Mus-TNF-α-F | GACGTGGAACTGGCAGAAGA |
| Mus-TNF-α-R | GGCTACAGGCTTGTCACTCG |
| Mus-IFNG-F | CGGCACAGTCATTGAAAGCC |
| Mus-IFNG-R | TGTTGTTGCTGATGGCCTGA |
| Mus-IL-1β-F | AGGAGAACCAAGCAACGACA |
| Mus-IL-1β-R | CTCTGCTTGTGAGGTGCTGA |
| Mus-CCL20-F | CGTCTGCTCTTCCTTGCTTTG |
| Mus-CCL20-R | CTGCTTTGGATCAGCGCAC |
| Mus-CXCL2-F | CTGGCCACCAACCACCAG |
| Mus-CXCL2-R | GCAAACTTTTTGACCGCCCT |
| Mus-GAPDH-F | TCACCATCTTCCAGGAGCGA |
| Mus-GAPDH-R | TTGCTGACAATCTTGAGTGA |
| Mer-IL-6-F | GATGGATGCTTCCATACTGGA |
| Mer-IL-6-R | GCCATTCCGTCTGTGACTC |
| Mer-TNF-α-F | GGAAGAGGCTTACGGATGGG |
| Mer-TNF-α-R | CAGCACAGACATGAAATCC |
| Mer-IFNG-F | CACACTGCATCTTGGCTT |
| Mer-IFNG-R | CTTTTGTGTCGCCATCCT |
| Mer-IL-1β-F | CAGCAGTGAAATGACAGCTT |
| Mer-IL-1β-R | CGGGCAAGAGACAGGCAT |
| Mer-CCL20-F | GCAGTGTCAAGCCTCTGCTC |
| Mer-CCL20-R | CACCCAGCTCTGCTTTGGAT |
| Mer-CXCL2-F | TCAGTGCCTGAAGACCCTAC |
| Mer-CXCL2-R | AATCTTTTGGATGATCCTCTG |
| Mer-GAPDH-F | GTGGCAAAGTGGAGATTGT |
| Mer-GAPDH-R | GAAGACACCGGTGGACTCC |
