# Supplementary material for: Giardia duodenalis MIF induces host intestinal damage via CD74 receptor mediated NLRP3 inflammasome activation
Source: PLoS Negl Trop Dis. 2026 Feb 2;20(2):e0013968. doi: 10.1371/journal.pntd.0013968 (PMC12880751; doi:10.1371/journal.pntd.0013968)
Supplement: S2 Data — (DOCX) [file pntd.0013968.s005.docx]

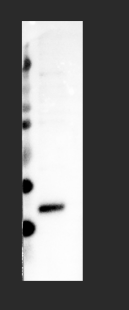
Fig1E

Fig2B


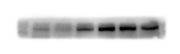
NLRP3
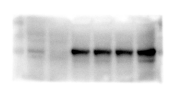
IL-1β
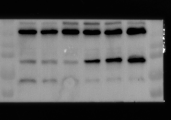
GSDMD


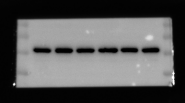
GAPDH
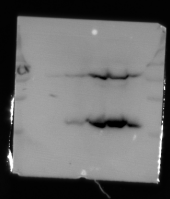
Caspase-1 p20
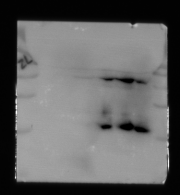
IL-1βp17

Fig2F


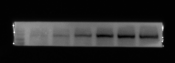
NLRP3
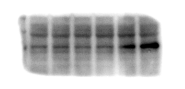
IL-1β
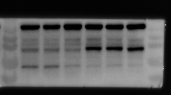
GSDMD


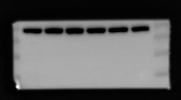
GAPDH
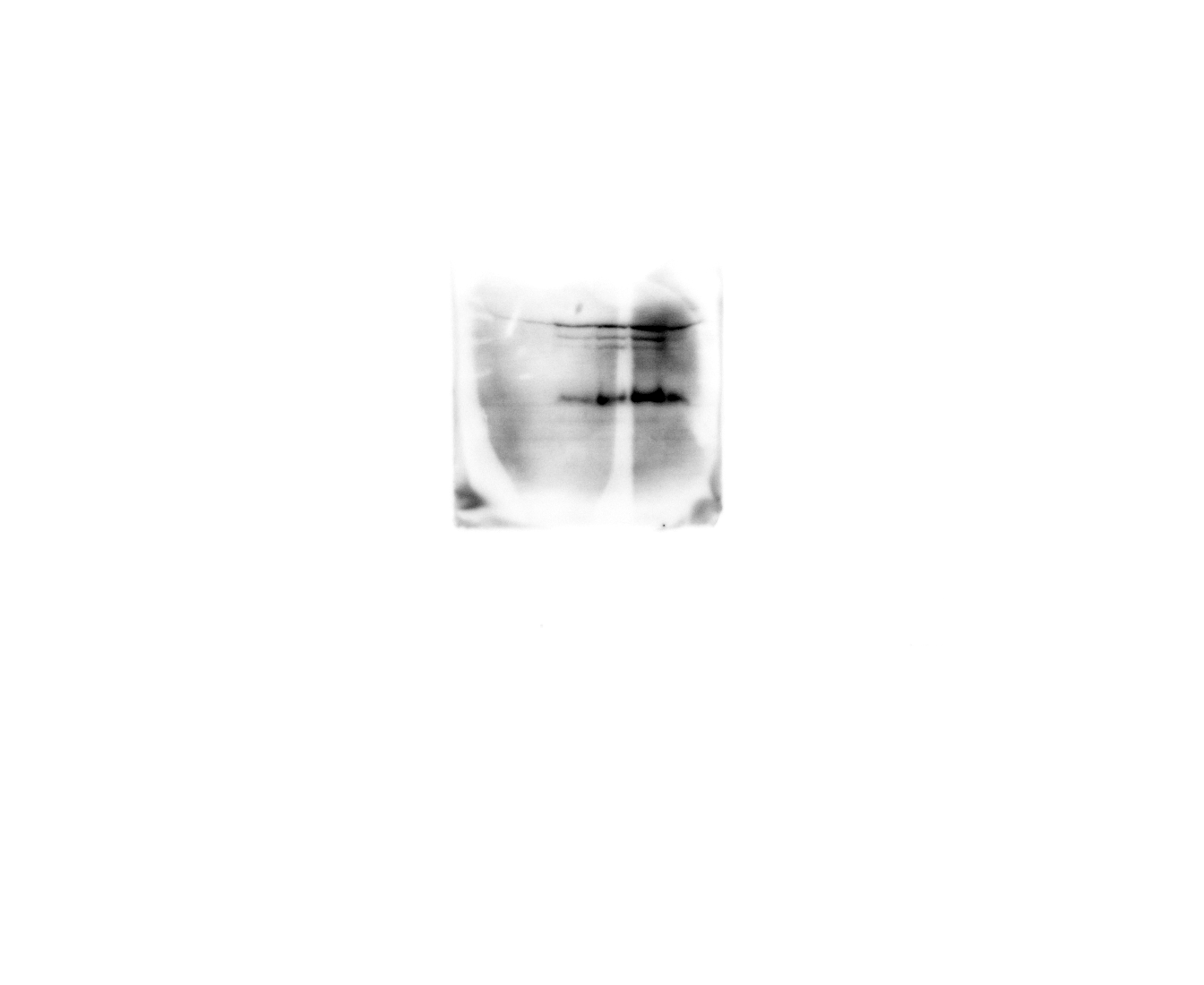
Caspase-1 p20
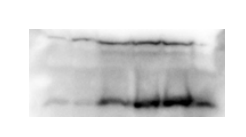
IL-1βp17

Fig3A


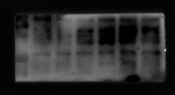
CD74-C
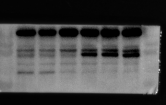
CD74-H


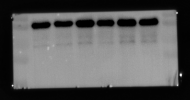
GAPDH-C
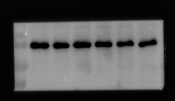
GAPDH-H

Fig3E

**
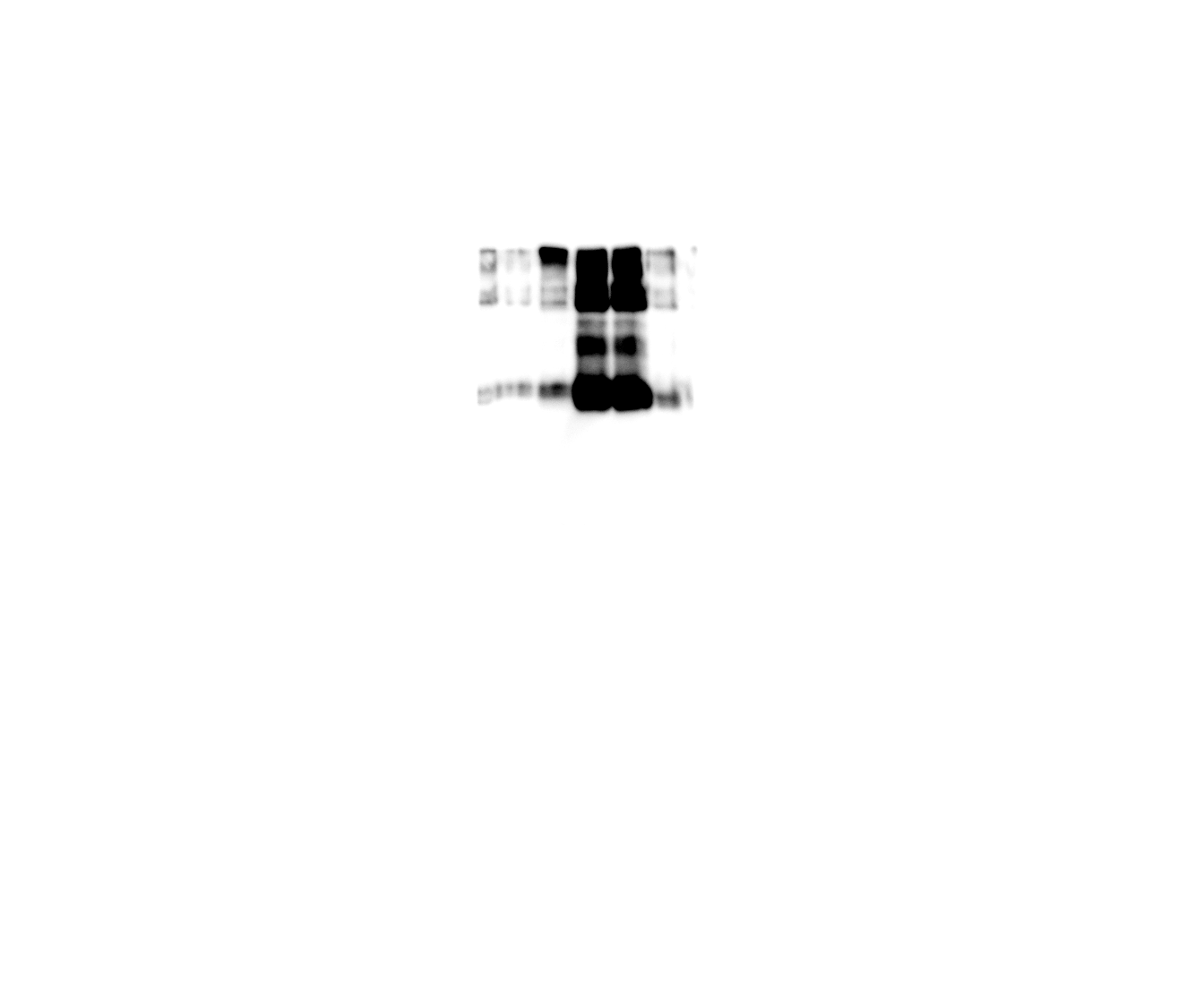
**IP:anti-HA **
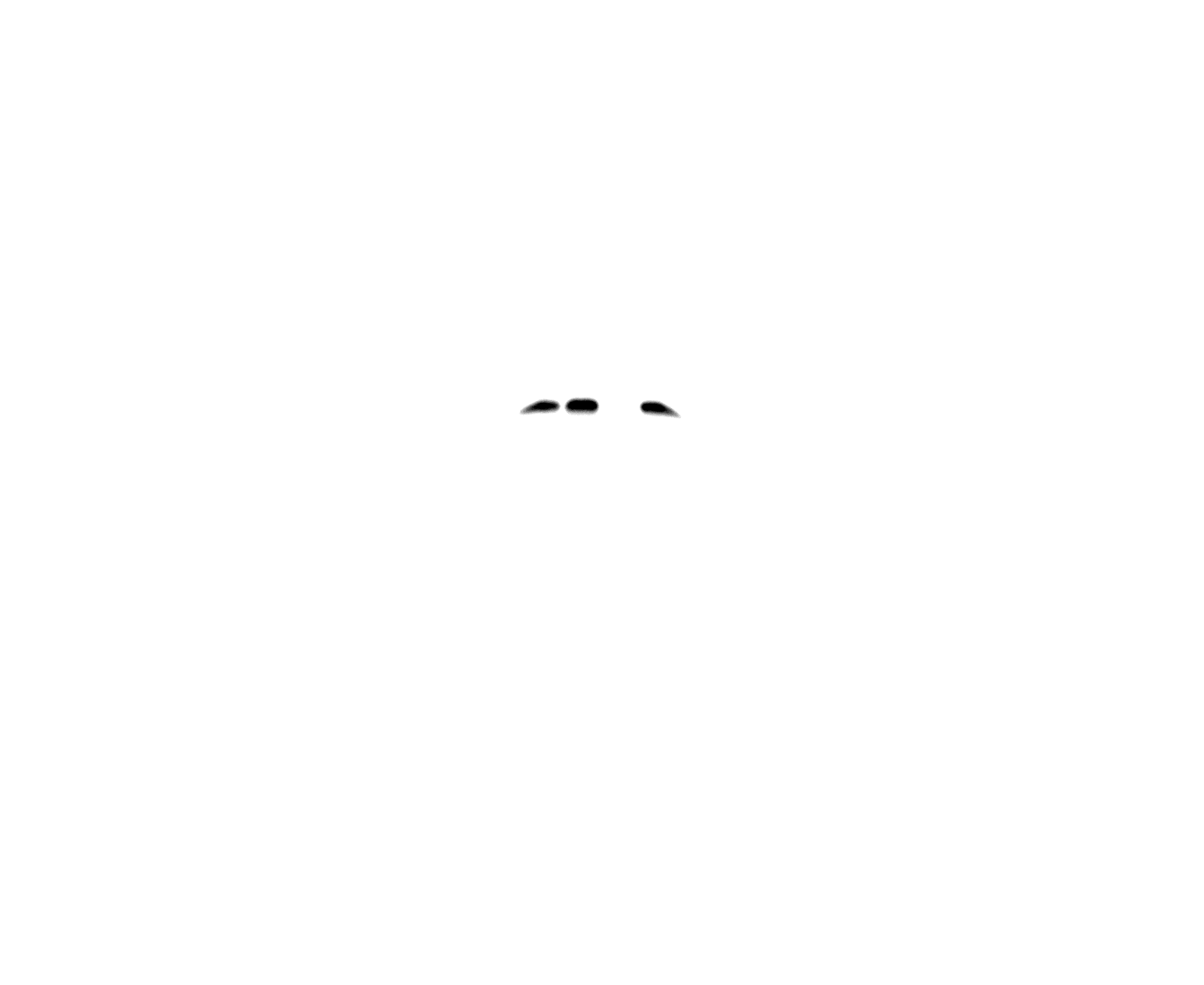
**input:anti-HA

**
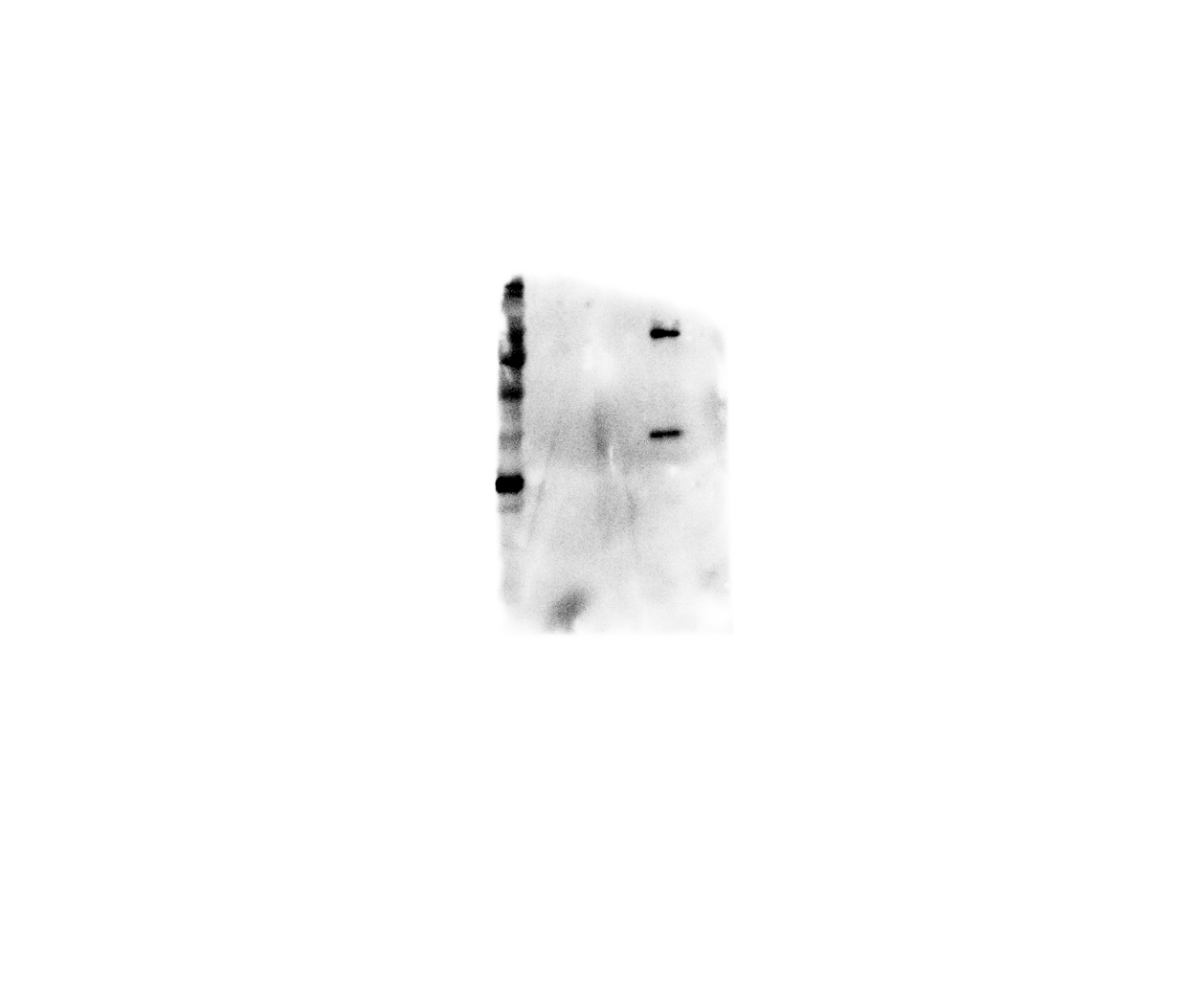
** IP:anti-His **
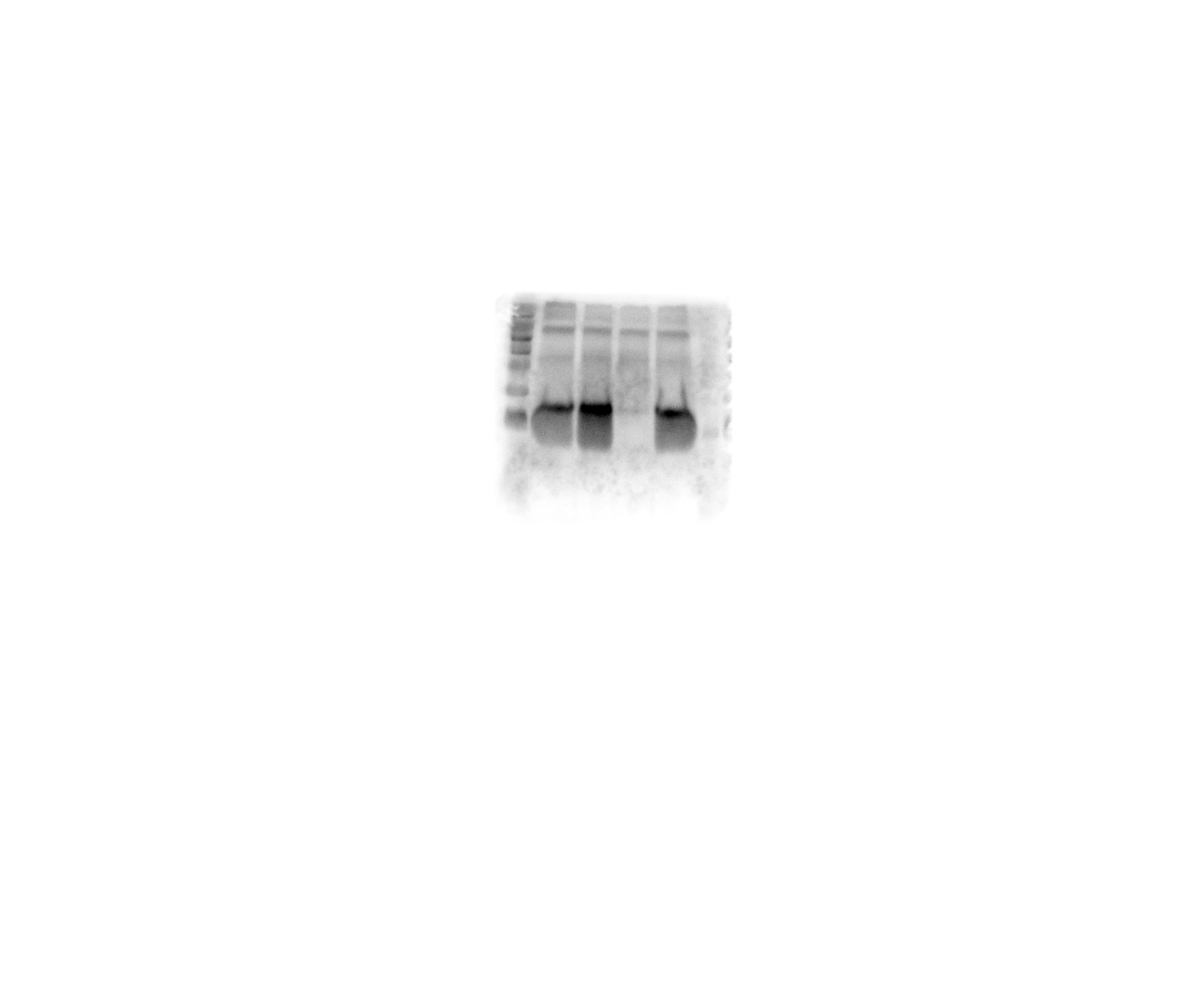
** input:anti-His

Fig4A


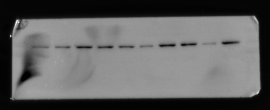
CD74
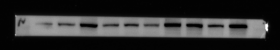
NLRP3
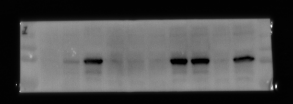
IL-1β


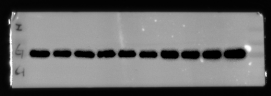
GAPDH
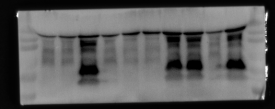
Caspase-1 p20
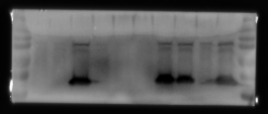
IL-1βp17

Fig4F


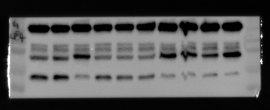
GSDMD
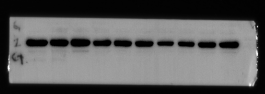
GAPDH

Fig5A


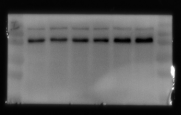
P-IKB
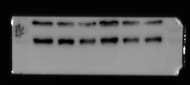
T-IKB
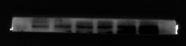
P-P65
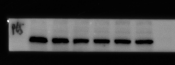
T-P65


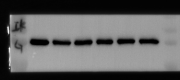
GAPDH

Fig5E


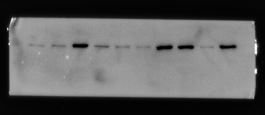
CD74


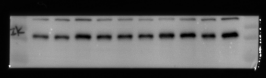
P-IKB
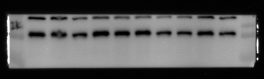
T-IKB


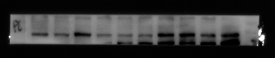
P-P65
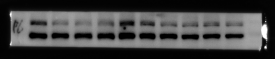
T-P65


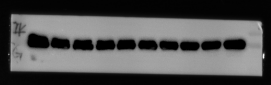
GAPDH

Fig5I


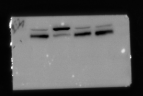
P-IKB
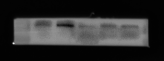
NLRP3
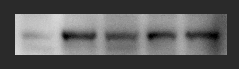
IL-1β


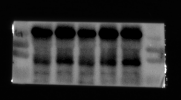
GSDMD
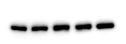
GAPDH


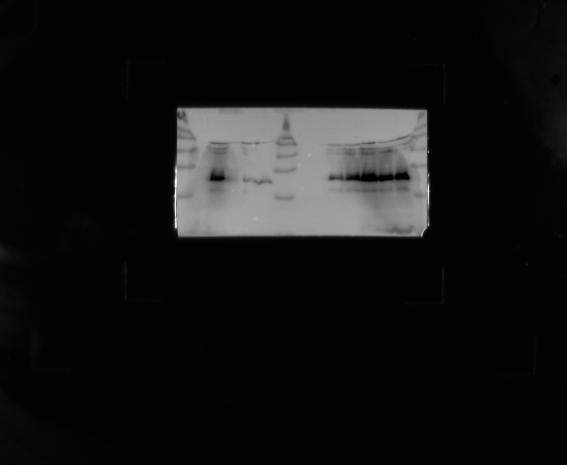
Caspase-1 p20
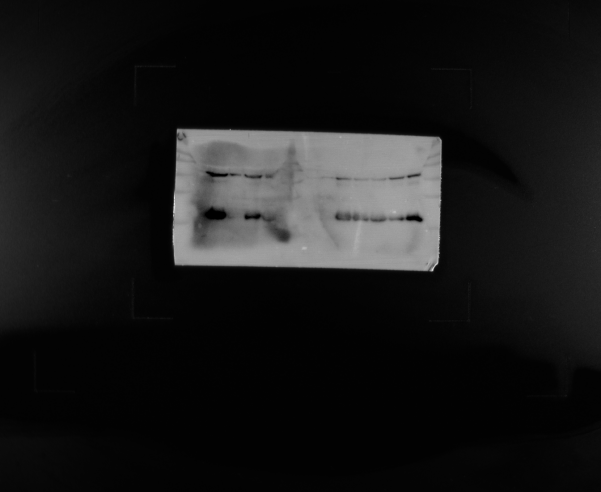
IL-1βp17

Fig7A


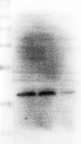


Fig7G


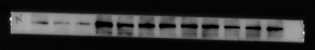
NLRP3
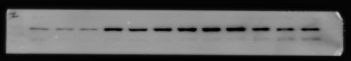
IL-1β


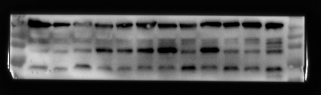
GSDMD
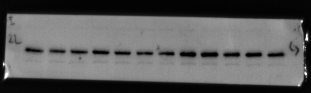
GAPDH

Fig8J


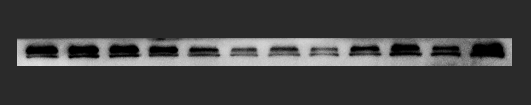
ZO-1
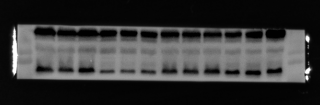
Occludin


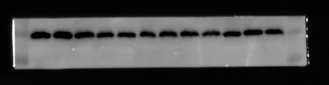
Claudin3
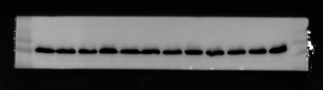
GAPDH

Fig9K


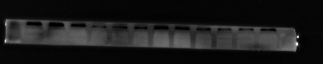
ZO-1
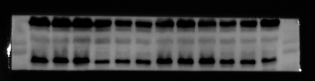
Occludin


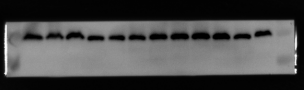
Claudin3
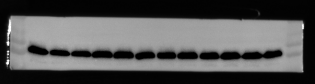
GAPDH

S1


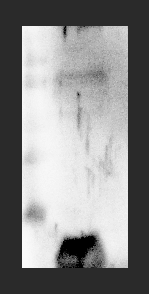
GdMIF antibody
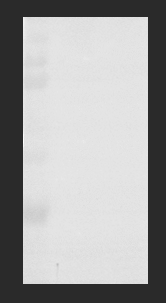
NC antibody
